# Mental health and well-being from childhood to adulthood: design, methods and results of the 11-year follow-up of the BELLA study

**DOI:** 10.1007/s00787-020-01630-4

**Published:** 2020-09-12

**Authors:** Christiane Otto, Franziska Reiss, Catharina Voss, Anne Wüstner, Ann-Katrin Meyrose, Heike Hölling, Ulrike Ravens-Sieberer

**Affiliations:** 1 Department of Child and Adolescent Psychiatry, Psychotherapy, and Psychosomatics, University Medical Center Hamburg-Eppendorf, Martinistr. 52, 20246 Hamburg, Germany; 2 Clinical Psychology, Helmut-Schmidt-University, Holstenhofweg 85, 22043 Hamburg, Germany; 3 Robert Koch-Institute, Nordufer 20, 13353 Berlin, Germany

**Keywords:** Health-related quality of life, Health care use, Children and adolescents, Young adults, Nation-wide survey, Longitudinal analyses

## Abstract

Mental health and well-being are of great interest in health policy and research. Longitudinal surveys are needed to provide solid population-based data. We describe the design and methods of an 11-year follow-up of the German BELLA study in children, adolescents and young adults, and we report on age- and gender-specific courses of general health and well-being, long-term health-related outcomes of mental health problems, and mental health care use. The BELLA study is the module on mental health and well-being within the German Health Interview and Examination Survey for Children and Adolescents (KiGGS). Standardised measures were used at each of the five measurement points of the BELLA study. In the 11-year follow-up, young people aged 7–31 years participated (*n* = 3492). Individual growth modelling, linear regression and descriptive analyses were conducted. Self-reported general health and well-being were both better in younger (vs. older) and in male (vs. female) participants according to the data from all five measurement points. Mental health problems in childhood and adolescence (measured at baseline) predicted impaired health outcomes at 6-year and 11-year follow-ups. Approximately one out of four children with a diagnosed mental disorder was not undergoing mental health treatment. With its 11-year follow-up, the prospective longitudinal BELLA study provides new and solid data on mental health and well-being from childhood to adulthood in Germany, and these data are important for health promotion and prevention practices. These results are consistent with previous findings. Promising future analyses are planned.

**Electronic supplementary material:**

The online version of this article (10.1007/s00787-020-01630-4) contains supplementary material, which is available to authorized users.

## Introduction

Mental health problems are the leading cause of health-related disability in children and adolescents worldwide [1] and are a global health challenge of the twenty-first century [2]. Likewise, mental health and well-being in childhood and adolescence have been the focus of interest among researchers in recent decades [3–5]. The magnitude of the problem also becomes clear when inspecting the global prevalence rates of mental disorders. Epidemiological studies report that approximately 13–20% of children and adolescents worldwide are affected by mental health problems [3, 6–8]. The results from a meta-analysis of 33 cross-sectional and longitudinal studies (*n* = 72,978) demonstrate that the overall prevalence of behavioural and emotional disorders among children and adolescents in Germany is 17.6% [9]. Research results from the representative 4 decade longitudinal birth cohort in New Zealand (Dunedin Study) and other longitudinal studies indicate that experiencing a diagnosable mental disorder at some point during the life course affects the majority of people rather than only a small subgroup [10, 11]. In the Dunedin cohort study, only 17% of the participants had never been diagnosed with a mental disorder between birth and midlife [10].

Mental health problems cause a high burden for both individuals and society and cause significant impairments in various life domains, such as family life, professional life, quality of life and the wider social environment [12–14]. From an economic perspective, mental disorders lead to high direct and indirect costs for society [15].

Mental disorders in children and adolescents are highly recurrent and persistent, and the development of comorbidities as well as chronic impairments during adulthood is frequent [6, 16–19]. With regard to health care utilisation, recent reviews point out that a number of barriers, such as structural issues, a lack of knowledge and understanding as well as negative attitudes towards mental health treatments, hinder affected children and adolescents or their parents from accessing mental health services [20, 21]. Overall, study findings underline the high relevance of mental health as an important factor in strengthening healthy childhood development and ensuring social participation.

Especially with regard to prevention and intervention, subjective well-being and health-related quality of life (HRQoL) are important aspects of modern concepts of health. HRQoL is a subjective and multidimensional construct that has become a major issue in epidemiological and clinical research and paediatric health care. The concept of HRQoL includes physiological, psychological, and functional aspects of health and well-being [22]. Recent research reviews underline that HRQoL and mental health problems are closely linked to each other, whereby children with mental health problems experience a noticeable reduction in various domains of HRQoL [4, 23]. Therefore, HRQoL measures can increase understanding the impact of mental health problems on children’s and adolescents’ lives and well-being and provide useful information for planning prevention and intervention strategies targeted to this age group [24].

The prospective longitudinal BELLA study focuses on mental health and well-being in children and adolescents in Germany and is conducted in close cooperation with the German Health Interview and Examination Survey for Children and Adolescents (KiGGS) of the Robert Koch Institute (RKI, Federal Public Health Institute of Germany). The BELLA study provides not only representative cross-sectional results on mental health and well-being in children and adolescents aged 7–17 years in Germany including information on mental health care use, but also longitudinal findings on developmental trajectories and on risk and protective factors of mental health and well-being from childhood via adolescence to young adulthood. The BELLA study is thus of high importance for public health and epidemiological research, and for research on resilience, as well as for health policy supporting the provision of targeted health care services, prevention and early intervention measures, and health promotion. The BELLA study has gathered data since 2003 at five measurement points using standardised and established measurement instruments. Data from the most recent 11-year follow-up (2014–2017) of the BELLA study are now ready to be analysed.

The present paper has the following objectives: first, to describe the design and methods of the 11-year follow-up of the longitudinal BELLA study, including non-response and dropout analyses; second, to report on age- and gender-specific courses of self- and parent-reported general health and HRQoL; third, to examine the long-term health-related outcomes of mental health problems during childhood and adolescence; and fourth, to report on mental health care use in children, adolescents and young adults in Germany.

## Methods

### Study design

**Fig. 1:**
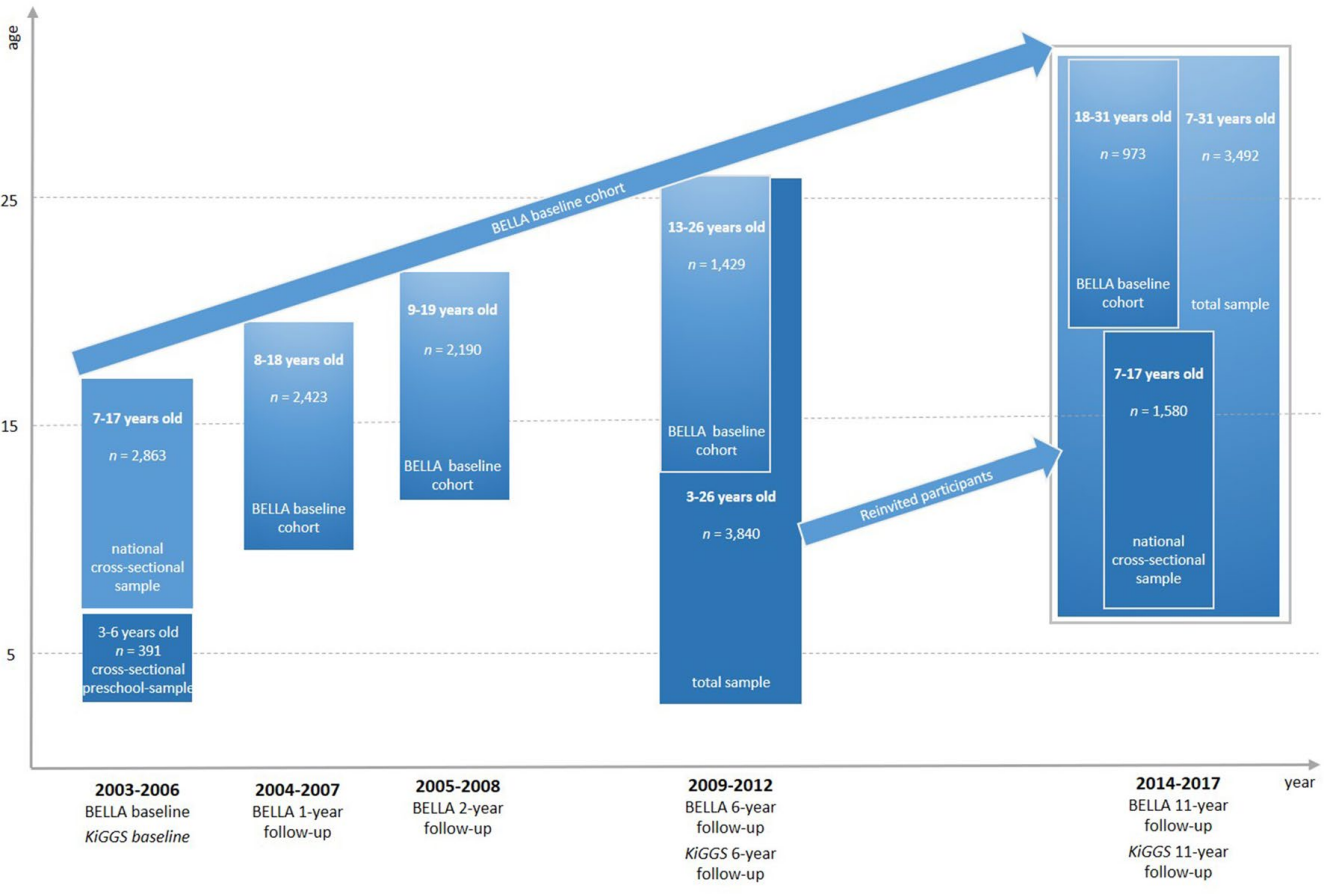
Measuring points of the BELLA study

The BELLA study is the module on mental health and HRQoL within the German Health Interview and Examination Survey for Children and Adolescents (KiGGS). Both studies have been conducted in close cooperation nationwide since 2003 and provide representative cross-sectional health- and mental health-related data on German children and adolescents as well as longitudinal data following participants into adulthood. The BELLA study uses a subsample of KiGGS. Participants were randomly drawn from the KiGGS sample and assigned to the BELLA study. The BELLA baseline assessment took place between 2003 and 2006 (*n* = 2863 children and adolescents aged 7–17 years) and was followed up at four measurement points, i.e., the 1 year (2004–2007), 2 year (2005–2008), 6 year (2009–2012), and the most recent 11-year follow-ups (2014–2017). New participants were included at the last two follow-ups to re-establish representative cross-sectional samples of children and adolescents and to compensate for loss due to dropout. Detailed information on the design of the BELLA study is presented in Fig. 1 (including a small preschool sample at BELLA baseline). Detailed descriptions of the KiGGS study [25, 26] and on the baseline assessment and first three measurement points of the BELLA study have been published [19, 27].

Only participants of KiGGS Wave 2 who agreed in KiGGS to be contacted for the BELLA study were invited to the 11-year follow-up. A letter was sent out including study information and a form to gather written informed consent. All participants and/or their parents were informed about the study procedures, told about means taken to protect their data and informed that participation was voluntary. Informed consent was gathered from the parents of children and adolescents younger than 18 years and from adolescents and young adults aged at least 14 years (adults could give their informed consent online as well). Data assessment was conducted online for the first time in the BELLA study; only if participants had no access to the internet or were not willing to participate online, a paper version of the questionnaire was provided (previous data assessments had been conducted by paper pencil questionnaires and computer-assisted telephone interviews). Parent reports were gathered on children aged 7–13 years, and self-reports were gathered in children, adolescents and young adults aged 11–31 years. The 11-year follow-up of the BELLA study was approved by the Federal Commissioner for Data Protection and received a positive vote from the Ethics Committee of Hamburg’s Chamber of Psychotherapists (on 24 September 2014).

### Sampling

**Fig. 2:**
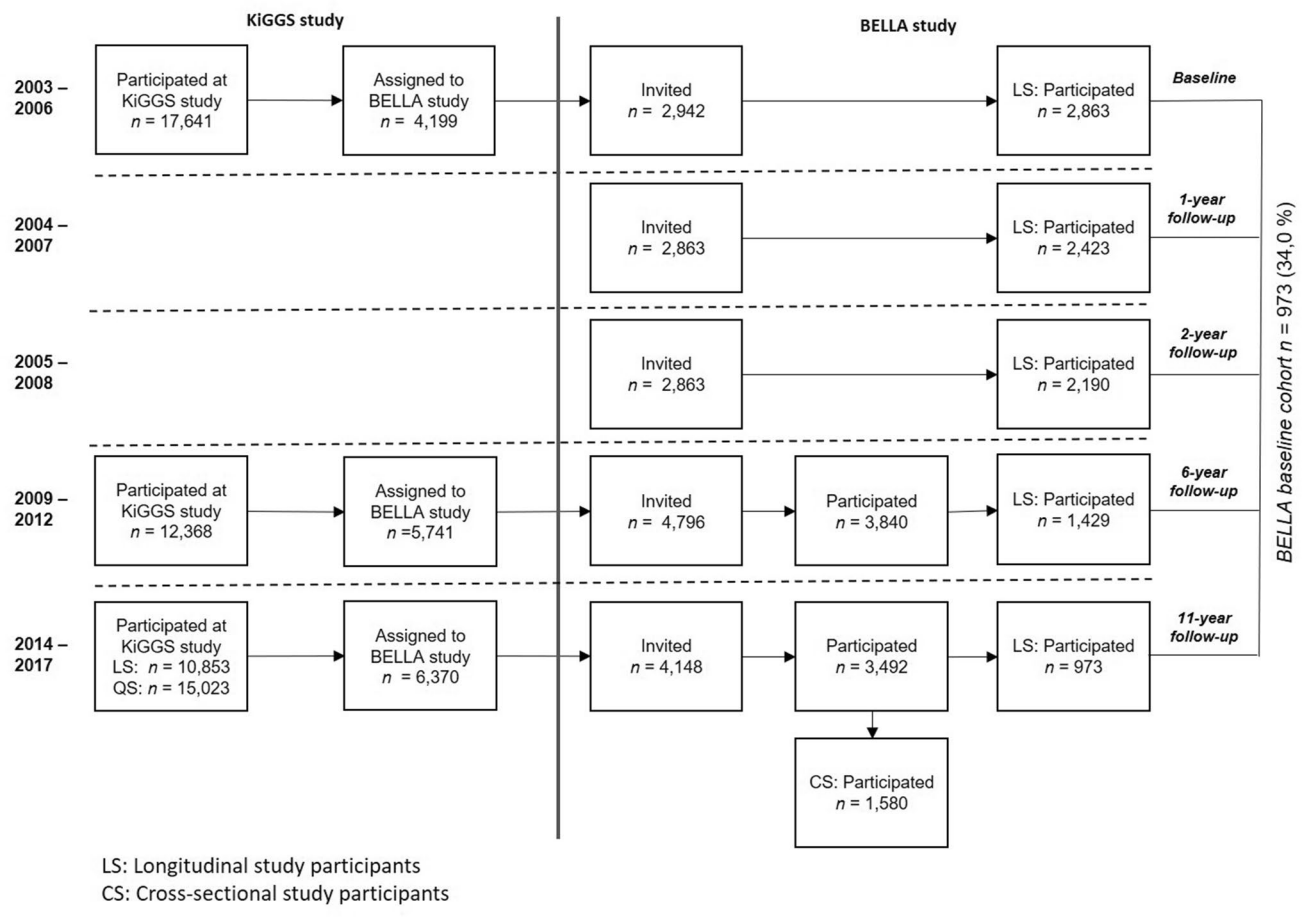
Numbers of invited and participating children and adolescents in the BELLA study

Participation in the most recent 11-year follow-up of the BELLA study required participation in KiGGS wave 2. The sampling for KiGGS wave 2 was conducted in two steps. First, cross-sectional sampling included randomly selected children and adolescents from 167 cities and municipalities in Germany, which were selected from official residency registries [28]. Second, for the longitudinal sampling in the KiGGS study, only participants who took part in the baseline assessment were followed up at KiGGS wave 2; KiGGS baseline participants could be included in KiGGS wave 2, if they agreed to participate [26]. Participants were excluded from the sample as quality neutral losses when they did not belong to the target population (e.g., invalid address, moved to a foreign country, deceased) or if communication with parents was not possible due to language barriers [28]. The numbers of invited and participating children and adolescents across all measurement points of the BELLA study are presented in Fig. 2. For the 11-year follow-up of the BELLA study, participants of the BELLA baseline assessment were re-invited for the baseline cohort sample of the BELLA study. In addition, new participants were included out of a randomly drawn subsample of the KiGGS wave 2 sample to allow representative cross-sectional analyses for children aged 7–17 years to be included in the cross-sectional sample. Please note, participants who participated for the first time in the BELLA study at the 6-year follow-up were not systematically re-invited for the 11-year follow-up, but the sampling procedure conducted in the KiGGS study resulted in a corresponding subsample in the BELLA study (see Fig. 2 and “Participants”). Out of the KiGGS wave 2 participants with an assignment to the BELLA study (*n* = 6370), 65.1% (*n* = 4148) agreed to be contacted by the BELLA study (baseline cohort sample: 73.7%; cross-sectional sample: 53.2%). Finally, *n* = 3492 children, adolescents and young adults participated in the 11-year follow-up of the BELLA study.

#### Response and cooperation rates

Response rates (RRs) and cooperation rates (COORs) were calculated according to the formulas RR2 and COOR 2 provided by the American Association for Public Opinion Research (AAPOR, [29]). Both rates were calculated twice, referring to the KiGGS participants with an assignment to the BELLA study (*n* = 6370) and regarding those KiGGS participants who agreed to be contacted by the BELLA study (*n* = 4148). Focusing on the latter sample, we calculated the response rate as the number of cases with valid survey data (*n* = 3492) divided by all cases we tried to get in contact with (i.e., those who participated, refused to participate, did not react at all to our invitation and we couldn’t reach via phone, and those with invalid contact information according to back-coming information); for calculating the corresponding cooperation rate, we divided the number of all cases with valid survey data by the number of cases we got in contact with (i.e., those who participated and those who refused to participate). The rates were calculated accordingly referring to the sample of KiGGS participants with an assignment to the BELLA study (using the corresponding numbers provided to us by the KiGGS study team). Of all KiGGS wave 2 participants assigned to the BELLA study (*n* = 6370), the (minimum) response rate was 56.5% and the cooperation rate was 68.7%. Of the families who had participated in KiGGS wave 2 and agreed to be contacted again by the BELLA study (*n* = 4148), *n* = 3492 finally participated in the 11-year follow-up with a (minimum) response rate of 84.5% and a cooperation rate of 94.5%.

#### Response analyses for participants and non-participants

Of those who agreed to be contacted by the BELLA study (*n* = 4148), *n* = 656 did not participate (15.8%). The main reasons were unavailability (66.2%, *n* = 398), active refusal (33.8%, *n* = 203), exclusion due to data quality issues (6.1%, *n* = 40) and quality neutral losses (i.e., letter undeliverable; 2.3%, *n* = 15). Among those young people who actively refused study participation, the main reasons were no interest (36.5%), no time (19.2%), and other reasons, e.g., negative experiences with studies or privacy issues (14.4%); approximately one-quarter of those who actively refused participation stated no reasons for refusal (23.7%), and a few people immediately hung up the phone when called to remind them of the study (7.4%).

Differences between the population of children and adolescents in Germany and KiGGS wave 2 participants are described elsewhere [26, 28]. We compared responders and non-responders of the KiGGS wave 2 participants with an assignment to the BELLA study (*n* = 6370). For this purpose, we predicted participation in the BELLA study by means of logistic regression analyses using sociodemographic (i.e., gender, age, urbanization, region, migration background, and SES) and health- and mental health-related variables (i.e., self- and parent-reported mental health problems, general health, physical health, impairments due to mental and physical health problems, and mental health care use in the last 12 months). To interpret our results, we followed recommendations [30] suggesting that OR = 1.68, 3.47, and 6.71 are equivalent to Cohen’s *d* = 0.2 (small), 0.5 (medium), and 0.8 (large), respectively. Only for age did we found a small effect, indicating that participation in the BELLA study was more likely in those aged 18–31 years than in those aged 14–17 years (OR = 1.73, 95% CI 1.51–1.97). For the remaining sociodemographic, health- and mental health-related variables, any effects were negligible.

#### Weighting

For the cross-sectional sample (at the 11-year follow-up), a weighting procedure was applied to ensure adaptation to the KIGGS wave 2 population. The KIGGS wave 2 cross-sectional sample was itself weighted to be representative of the population in Germany taking the survey design (selection of a particular sample point and selection of participants within the sample point) and population distributions regarding age, gender, federal state (as of 31 December 2015) and foreigner status (German nationality yes/no; as of 31 December 2014) into account [28]. For the BELLA cross-sectional sample, a weighting variable was calculated based on two steps: (1) the inverse participation probability multiplied by the KIGGS wave 2 weight was calculated based on the best participation probability model for participation in the BELLA study considering age, gender, citizenship of the mother, SES, current smoking of the mother, community size, highest education status of the parents, and apartment size; (2) an adaption weight was calculated to ensure comparability with the abovementioned population distributions covering four levels, namely, (i) age x gender, (ii) region (West, Berlin, East) × age group × education status of the parents, (iii) federal state × gender × age group, and (iv) region (West incl. Berlin vs. East) × foreigner status.

#### Dropout analyses for the 11-year follow-up

Regression analyses were conducted to examine systematic dropout at the 11-year follow-up for participants of the BELLA baseline (*n* = 2863) using sociodemographic and health- and mental health-related variables. Small effects found in the baseline sample indicated that dropout at the 11-year follow-up was more likely among those with a lower SES than a moderate SES (OR = 2.66, 95% CI 1.99–3.56) and with non-German citizenship (OR = 2.35, 95% CI 1.54–3.59). For remaining sociodemographic, health- and mental health-related variables, effects were negligible, if found at all.

### Participants

Based on the 11-year follow-up, the BELLA sample can be differentiated into three main samples (see Fig. 2): first, a cross-sectional sample (*n* = 1580) including children aged 7–17 years, who were randomly selected for each age category and represent the German population for this age group; second, the baseline cohort sample of *n* = 973 participants of BELLA baseline (34.0% out of *n* = 2863 baseline participants); third, a total sample of all participants at the 11-year follow-up BELLA study (*n* = 3492) including those who had already participated at the 6-year follow-up, but not at previous measurement points of the BELLA study (*n* = 1050; 43.6% out of *n* = 2411 new 6-year follow-up participants). The sampling procedure conducted by the KiGGS study in combination with the fact that some villages used as sample points in the KiGGS study only had very small numbers of inhabitants, resulted in the following situation for the BELLA study. One individual from the baseline cohort sample and *n* = 110 individuals from the total sample, who had participated already and for the first time at the 6-year follow-up of the BELLA study, were included in the cross-sectional sample as well. The total sample of the 11-year follow-up of the BELLA study thus includes *n* = 3492 individuals (3603 cases summarized over all three samples minus 111).

Details on the sociodemographic characteristics of the BELLA cross-sectional sample (weighted and unweighted data), the BELLA baseline cohort sample (unweighted) and the BELLA total sample (unweighted) at the 11-year follow-up are presented in Table 1. The sociodemographic characteristics, region, migration background, and SES were almost equally distributed across all unweighted samples at the 11-year follow-up (please note, SES was measured in children and adolescents younger than 18 years based on information on income, profession and education of the parents). Participants in the BELLA baseline cohort sample were older (*M* = 23.17, SD = 3.32) at the 11-year follow-up compared to those in the BELLA total sample (*M* = 17.33, SD = 5.83) and those in the BELLA cross-sectional sample (*M* = 13.02, SD = 2.94). The age of participants at each measurement point of the BELLA study can be found in the Supplementary Material (File 1, Table S1).

**Table 1:** Socio-demographic characteristics of the BELLA sample at the 11-year follow-up

	BELLA cross-sectional sample, weighted (*n* = 1580)^a^			BELLA cross-sectional sample, unweighted (*n* = 1580)			BELLA baseline cohort sample, unweighted (*n* = 973)			BELLA total sample, unweighted (*n* = 3492)		
	*n*	%	*M* (SD)	*n*	%	*M* (SD)	*n*	%	*M* (SD)	*n*	%	*M *(SD)
Gender												
Male	*816*	*51.6*		754	47.7		389	40.0		1550	44.4	
Female	*764*	*48.4*		826	52.3		584	60.0		1942	55.6	
Age			*12.73 (3.21)*			13.02 (2.94)			23.17 (3.32)			17.33 (5.83)
7–10 years	*545*	*34.5*		442	28.0		–	–		442	12.7	
11–13 years	*409*	*25.9*		514	32.5		–	–		779	22.3	
14–17 years	*626*	*39.6*		624	39.5		3	0.3		881	25.2	
18–31 years	–	–		–	–		970	99.7		1390	39.8	
Region												
Eastern Germany (incl. Berlin)	*267*	*16.9*		544	34.4		335	34.4		1184	33.9	
Western Germany	*1313*	*83.1*		1,036	65.6		638	65.6		2308	66.1	
Community size (inhabitants)												
Rural (< 5000)	*283*	*17.9*		383	24.2		157	16.1		689	19.7	
Small town (5000– < 20,000)	*446*	*28.2*		459	29.1		225	23.1		925	26.5	
Medium-sized town (20,000– < 100,000)	*477*	*30.2*		419	26.5		242	24.9		944	27.0	
Metropolitan (≥ 100,000)	*374*	*23.7*		319	20.2		349	35.9		934	26.7	
Migration background												
No	*1200*	*76.0*		1379	87.3		868	89.2		3048	87.3	
One sided	*160*	*10.1*		113	7.2		42	4.3		221	6.3	
Two sided	*210*	*13.3*		79	5.0		63	6.5		210	6.0	
No information	*10*	*0.6*		9	0.6		–	–		13	0.4	
Living situation (family of origin, own family)												
Living with both biological parents	*1223*	*77.4*		1276	80.8		299	30.7		2160	61.9	
Living with one biological parent^b^	*306*	*19.4*		279	17.7		100	10.3		540	15.5	
Living with a partner	–	–		–	–		268	27.5		350	10.0	
Living alone	–	–		–	–		134	13.8		191	5.5	
Living in a flat-sharing community	–	–		–	–		101	10.4		131	3.8	
Living in another family structure^c^	*30*	*1.9*		17	1.1		18	1.8		45	1.3	
No information	*21*	*1.3*		8	0.5		53	5.4		75	2.1	
Socio-economic status (family of origin)			*12.83 (3.91)*			14.35 (3.84)			–			14.29 (3.87)
Low	*264*	*16.7*		122	7.7		–	–		175	5.0	
Middle	*1013*	*64.1*		960	60.8		–	–		1318	37.7	
High	*274*	*17.4*		488	30.9		–	–		656	18.8	
No information	*28*	*1.8*		10	0.6		–	–		1343	38.5	

Weighted data in *Italics*

*M* = mean, SD = standard deviation

^a^Data weighted to correct for deviation of the sample from the age, gender, parental education, regional, citizenship, and foreigner status structure of the German population (in 2015); for details see sampling Section

^b^Living with one biological parent includes participants living with a single biological parent with or without a new partner and living in shared care

^c^Living in another family structure includes participants living with grandparents or other relatives, with adoptive or foster parents, and in children’s homes or boarding schools

### Measurements

For the 11-year follow-up of the BELLA study, data assessment was conducted mainly online; only if participants refused to fill out the online questionnaire or had no access to the internet was a paper version of the questionnaire provided. Self-reported data were collected from children and adolescents aged 11 years and older, and parent-reported data were additionally gathered for children younger than 14 years. The BELLA study used standardised instruments if available (complemented by self-developed measurements) to assess different aspects of health, HRQoL, mental health problems and mental health care utilisation. An overview of the instruments used across all measurement points of the BELLA study is provided in the Supplementary Material (File 1, Table S2). In addition, a large number of variables raised by the KiGGS study as indicators of somatic health (e.g., body mass index, blood pressure, laboratory parameters), health behaviour (e.g., nutrition, sports activities), and sociodemographic determinants (e.g., SES, migration background) are available and can be linked to mental health indicators from the BELLA study [31, 32]. We describe key measures administered at the 11-year follow-up in the following sections. Instruments used for analyses in the present article are mentioned again in the data analysis and results section (including information on their internal consistency in the corresponding samples under analysis).

#### Health and health-related quality of life

General health was assessed using the general health item (GHI) in self- and parent reports (“In general, how would you rate your/your child’s health?”) with a five-point response scale (1 = “excellent”, 2 = “very good”, 3 = “good”, 4 = “fair”, 5 = “poor”). The GHI is well-established and recommended by the WHO for use in health surveys [33]. To measure self-reported HRQoL, the Kids-CAT was administered for the first time in a large population-based epidemiological sample. The Kids-CAT tool, developed and validated by the authors of this article, measures HRQoL in healthy and ill children and adolescents based on the five item banks on physical well-being, psychological well-being, parent relations, social support and peers, and school well-being [34, 35]. Acceptable to good internal consistency was found for the Kids-CAT dimensions in its validation study (mean standard errors of measurement ranged from 0.38 to 0.49 corresponding to Cronbach’s alphas from 0.76 to 0.86, [35]). The IRT-based measurement selects and administers the most informative items for each participant based on his or her location on the underlying latent trait [36]. Therefore, the Kids-CAT provides fewer items and is as precise as traditional paper–pencil questionnaires. It has a child-friendly design and was easily accessible via the BELLA online questionnaire. For the first time, we also integrated a static proxy version of the most powerful Kids-CAT items to survey the parents’ perspective at the 11-year follow-up. Moreover, the well-established self- and parent-reported KIDSCREEN-27, including the KIDSCREEN-10 index with a five-point response scale (0 = “not at all” to 4 = “extremely” or 0 = “never” to 4 = “always”) [37], the SF-12 questionnaire [38], and the SF-36 questionnaire [39], were administered to measure HRQoL. Furthermore, validated short questionnaires of the item banks developed by the Patient-Reported Outcome Measurement Information System (PROMIS®) initiative [40, 41] were used to assess subjective well-being, family relations, physical activity, relations with peers, and global health. Good to mainly excellent internal consistency was reported for original PROMIS scales [42–49]. Within the scope of the BELLA study, the PROMIS questionnaires were translated into German (see e.g., [50], more publications on translations are to follow).

#### Mental health problems

At all measurement points, parent- and self-reports on mental health problems were assessed with the Strengths and Difficulties Questionnaire (SDQ) accompanied by the 5-item SDQ Impact supplement asking for difficulties that upset or distress the child and for interference with home life, friendships, classroom learning, and leisure activities with a four-point response scale (0 = “not at all”, 1 = “only a little”, 2 = “quite a lot”, 3 = “a great deal”) [51, 52]. For respondents aged 18 years and older, the Composite International Diagnostic-Screener (CID-S) [53] and the Symptom-Check List 9-item Short version (SCL-S-9) [54] were used at the 11-year follow-up. To survey symptoms of depression, the Center for Epidemiological Studies Depression Scale for Children and Adolescents (CES-DC, [55]) and the Patient Health Questionnaire-9 for Young Adults (PHQ [56, 57];) were used. Furthermore, depressive symptoms were assessed using the German translations of PROMIS Depression Short Forms across all age groups [58, 59]. The SCL-S-9, the CES-DC and the PHQ showed good to excellent internal consistency in former studies (Cronbach’s alphas ≥ 0.80 and 0.90, respectively; [54, 60, 61]).

#### Mental health care utilisation

Mental health service utilisation was assessed by surveying the psychiatric/sociopsychiatric/psychotherapeutic, psychological, or sociopedagogic care that respondents had used and how satisfied they had been with their treatment. Additionally, we assessed possible treatment needs and barriers that prevented people from accessing treatment.

### Data analysis

#### Age- and gender-specific effects on general health and health-related quality of life over time

We investigated age- and gender-specific effects on self- and parent-reports of general health measured with the GHI and on HRQoL assessed with the KIDSCREEN-10 index using all available data across the measurement points of the KiGGS and BELLA studies. For analyses, we recoded response options of the GHI so that higher values indicated better general health. We calculated *T* values (*M* = 50; SD = 10) for the KIDSCREEN-10 index based on Rasch Person parameters of the European norm sample [37], with higher values indicating better HRQoL. Individual growth modelling was used for data analyses calculating linear mixed models, which allowed for repeated measurements using full-information maximum likelihood (FIML). Each model included age (at baseline), gender, the interaction age by gender, a linear time variable (with information on intervals between baseline and the measurement point in question in years), a squared and a cubic time variable as fixed effects; on the level of random effects, a subject identification variable was considered as random intercept and linear time was used as random slope. For each model, age was centred using the group mean at baseline (across all participants with valid baseline scores*; M*_age,t0 valid_); for parent-reported HRQoL, the mean age from the 1-year follow-up was used (*M*_age,t1 valid_) since no corresponding baseline data were gathered. We created graphs to illustrate gender-specific trajectories across age based on data from all measurement points using estimated marginal means from corresponding models. In preliminary analyses, we investigated potential cohort effects for each outcome. Random intercept models served to investigate whether the year of birth moderated the relationship between age (at each measurement point) and the outcome in question. Since information criteria and the *χ*^2^ difference test depend on sample size [62], we used McFadden’s *R*^2^ [63] to evaluate the strengths of potential cohort effects comparing models with and without the interaction term of interest.

#### Mental health problems at baseline and related outcomes at 6-year and 11-year follow-ups

To examine the association between self- and parent-reported mental health problems (measured with the SDQ and SDQ Impact) reported at baseline and health-related outcomes at 6-year and 11-year follow-ups, we developed univariate general linear models for each perspective (self- and parent-reports at baseline), outcome (self-reported general health, mental health, physical health) and measurement point (6-year and 11-year follow-ups and). We included only predictors measured at baseline, i.e., mental health problems, impairment due to mental health problems (none, moderate, high), gender, age, SES, and the interaction of gender by age. Regarding health-related outcomes measured at 6-year and/or 11-year follow-up, we used the first item of the SF-36 to assess general health and transformed the item to a scale from 0 to 100, with higher scores indicating better general health; furthermore, the mental and physical health components of the SF-36 were used and standardised to a mean of 50, with a score above 50 representing better than average function and a score below 50 representing poorer than average function. Effect sizes were calculated using partial eta squared (*η*^2^ = 0.01 indicates a small, *η*^2^ = 0.06 a medium, and *η*^2^ = 0.14 a large effect).

#### Mental health care utilisation

Descriptive analyses were conducted on mental health care use and barriers to mental health care use.

All analyses were conducted with IBM SPSS 26.

## Results

### Age- and gender-specific effects on general health and health-related quality of life

Across all measurement points, valid data for self-reported general health were available for *n* = 4987 (52% female; overall, 10,213 valid scores were gathered across measurement points in 10- to 31-year-olds). With the parent-reported GHI, valid information was gathered in *n* = 5754 (50% female; overall 11,149 scores for 3- to 20-year-olds). Due to strong ceiling effects for both versions of the GHI (the option “poor” was chosen for less than 1% of the ratings for each version; self-report: *n* = 35; parent-report: *n* = 26), we collapsed response options (gathering “fair” and “poor”). The results from null models indicated that 37% of the total variance in the self-reported and 42% for the parent-reported general health score could be explained by differences between subjects. Fit information on null models and results of final models are depicted in the Supplementary Material (File 2, Tables S3 and S4). For self-reported general health, we found an overall mean of 2.80 after controlling for covariates in the final model (i.e., average score at baseline for boys aged approximately 14 years; *M*_age,t0 valid_ = 13.94, SD_age,t0 valid_ = 2.005). Effects for time variables indicated a slight increase over time. Better self-reported general health was found in younger than older and in male compared to female participants; the age-specific difference was more pronounced in girls. For parent-reported general health, an overall mean of 2.87 was estimated (at baseline for boys aged approximately 12 years; *M*_age,t0 valid_ = 11.75, SD_age,t0 valid_ = 3.145), and again, a slight increase over time was found. Parent-reported general health was overall better for younger than older participants, with higher scores for girls in younger participants and higher scores for boys in older participants. Findings from sensitivity analyses using general estimation equation models (GEEs) for categorical outcomes [64] were similar.

For HRQoL, valid self-reports were gathered with the KIDSCREEN-10 index in *n* = 4293 (51% female; overall 7136 scores from 10- to 20-year-olds), and parent-reports were gathered in *n* = 4345 (50% female; overall 6783 scores for 6- to 20-year-olds). Due to the results from the null models, 48% of the total variance in self-reported HRQoL and 49% for parent-reported HRQoL could be explained by differences between the subjects’ model fit results (Table S3). The internal consistency of the KIDSCREEN-10 was acceptable to good in the present study (Cronbach’s alphas ranged for the self-report from 0.78 to 0.82 and for the parent-report from 0.74 to 0.79 across measurement points in the analysed samples). The results of the final model for self-reported HRQoL (Table S4) showed an overall mean of 53.37 (at baseline for boys aged approximately 14 years; *M*_age,t0 valid_ = 13.94, SD_age,t0 valid_ = 1.984), a slight increase over time, and higher scores for younger than older and for male compared to female participants; the decrease with ongoing age was more pronounced in girls. For parent-reported HRQoL according to the KIDSCREEN-10 index, an overall mean of 53.55 was estimated (at 1-year follow-up for boys aged 12 to 13 years; *M*_age,t1 valid_ = 12.62, SD_age,t1 valid_ = 3.122), which increased slightly over time; higher scores were reported for younger than older participants with better HRQoL for girls in younger and for boys in older ones.

**Fig. 3:**
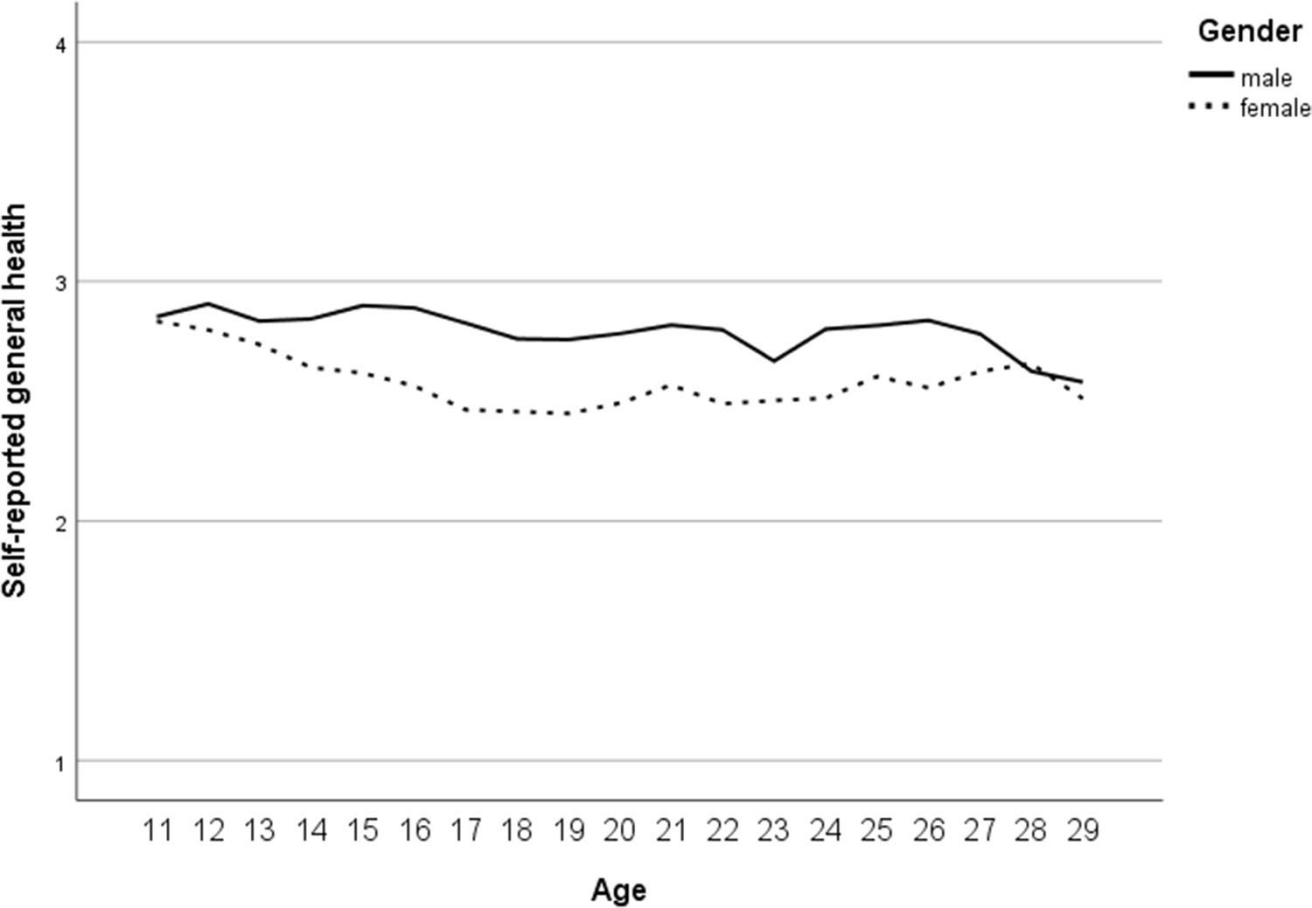
Gender-specific course of self-reported general health (according to the general health item; GHI) from age 11 to age 29 (1 = ‘poor’ / ‘fair’, 2 = ‘good’, 3 = ‘very good’ and 4 = ‘excellent’ general health)

**Fig. 4:**
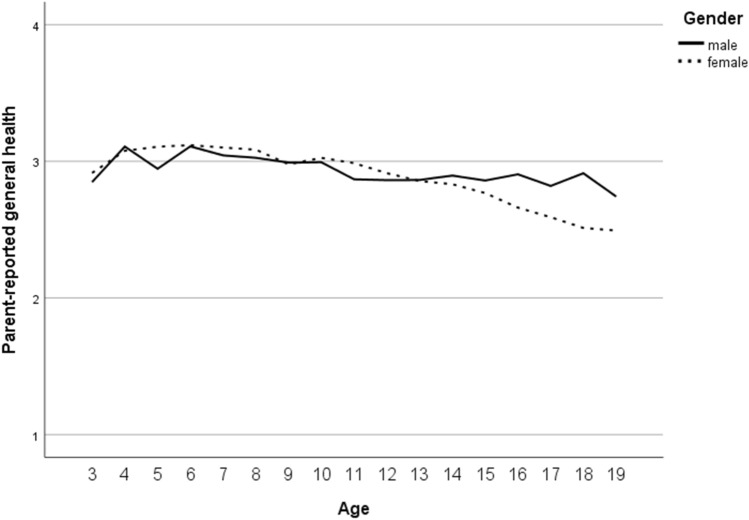
Gender-specific course of parent-reported general health (according to the general health item; GHI) from age 3 to age 19 (1 = ‘poor’ / ‘fair’, 2 = ‘good’, 3 = ‘very good’ and 4 = ‘excellent’ general health)

**Fig. 5:**
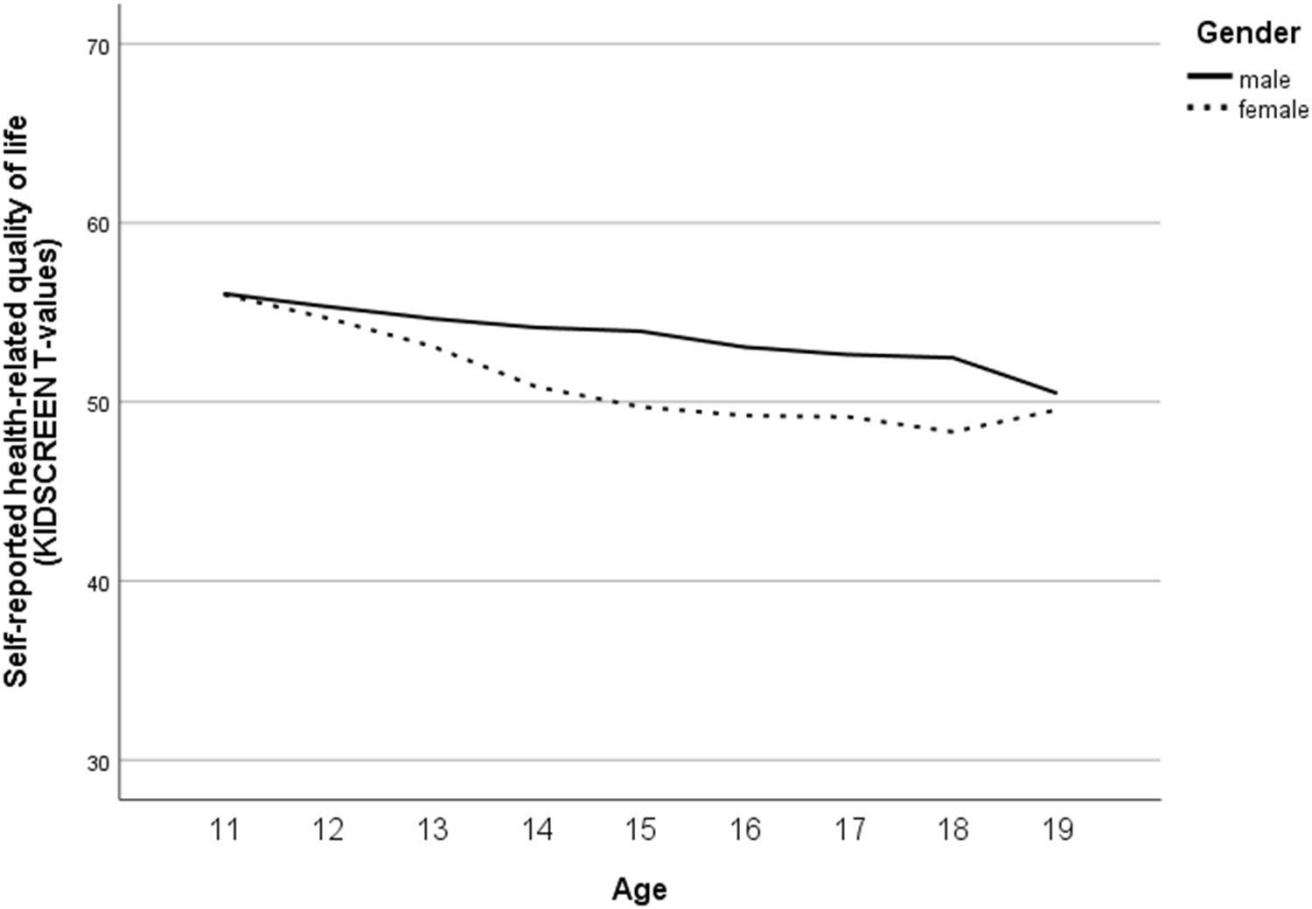
Gender-specific course of self-reported health-related quality of life (according to the KIDSCREEN-10 index) from age 11 to age 19

**Fig. 6:**
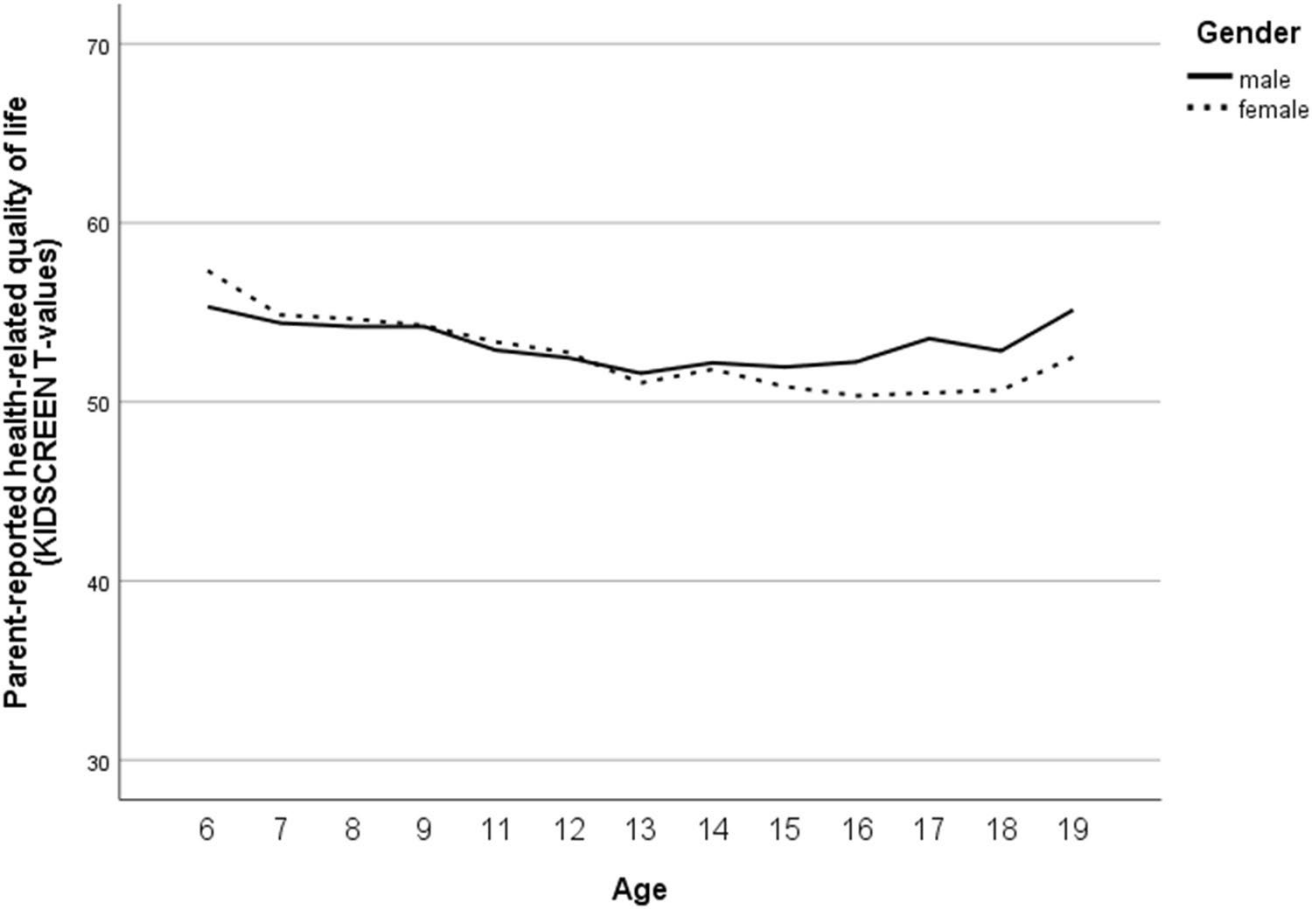
Gender-specific course of parent-reported health-related quality of life (according to the KIDSCREEN-10 index) from age 6 to age 19

In the presented final models (Table S4), random effects indicated significant differences in the variances of intercepts and slopes across participants, and only the variance of the slope for self-reported HRQoL was not significant. Intercepts and slopes covaried negatively and significantly; only for parent-reported HRQoL was a positive significant covariance found. In preliminary analyses, we further found no evidence for a cohort effect in any outcome; McFadden’s *R*^2^ was consistently below 1%, if corresponding effects were significant at all. Figures 3, 4, 5 and 6 present graphs on gender-specific courses across age (only for this, we collapsed age categories at margins, if a category was represented by less than *n* = 30), which reflect findings from the models as reported.

### Mental health problems at baseline and related outcomes at 6-year and 11-year follow-ups

We investigated associations of self- and parent-reported mental health problems in children and adolescence and their impact measured at baseline (using the SDQ and its impact supplement) with self-reported general, mental and physical health at 6-year and 11-year follow-ups (according to the SF-36). Based on self-reported baseline data, we analysed *n* = 566 with self-reported at the 6-year follow-up (females: *n* = 306; mean age at baseline: *M* = 14.11, SD = 1.86) and *n* = 504 at the 11-year follow-up (females: *n* = 315; mean age at baseline: *M* = 13.94, SD = 2.01). Based on parent-reported baseline data, we examined health outcomes of *n* = 597 at the 6-year follow-up (females: *n* = 316; mean age at baseline: *M* = 13.95, SD = 2.00) and *n* = 886 at the 11-year follow-up (females: *n* = 529; mean age at baseline: *M* = 11.69, SD = 3.17). The internal consistency of SDQ scores was consistently acceptable in the analysed samples (for the self-reported total difficulties score Cronbach’s alphas were 0.75 and 0.73, and for the self-reported impact supplement.70 and 0.74; for parent reports, alphas for the total score were 0.79 and 0.78, and for the impact supplement 0.76 and 0.75; please note the impact includes a key item and its internal consistency across all items is thus calculated based only on those with an impact of mental health problems). For the investigated SF-36 sum scores, the internal consistency was excellent in the analysed samples (Cronbach’s alphas were 0.90 for the 6-year follow-up and 0.94 for the 11-year follow-up).

**Fig. 7:**
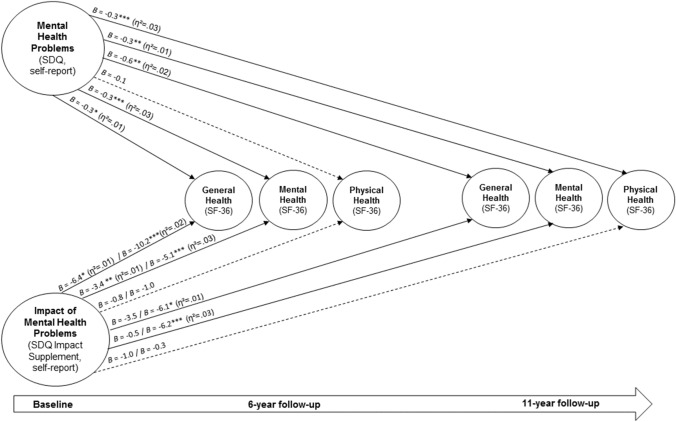
Long-term effects of self-reported mental health problems and of the impact of mental health problems (according to the Strengths and Difficulties Questionnaire (SDQ) and its impact supplement) at baseline on health-related outcomes (according to the SF-36) at 6-year and 11-year follow-ups. *B* = unstandardized regression coefficient; *p < 0.05, **p < 0.01, ***p < 0.001; dashed lines indicate non-significant effects, continuous arrows indicate significant effects; *η*^2^ indicates effect sizes; for the impact of mental health problems, groups borderline and abnormal were compared each to the group normal

**Fig. 8:**
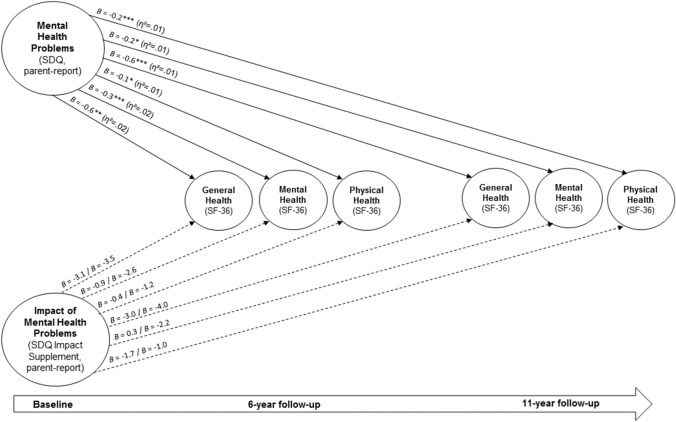
Long-term effects of parent-reported mental health problems and of the impact of mental health problems (according to the Strengths and Difficulties Questionnaire (SDQ) and its impact supplement) at baseline on health-related outcomes (according to the SF-36) at 6-year and 11-year follow-ups. *B* = unstandardized regression coefficient*;* *p < 0.05, **p < 0.01, ***p < 0.001; dashed lines indicate non-significant effects, continuous arrows indicate significant effects; *η*^2^ indicates effect sizes; for the impact of mental health problems, groups borderline and abnormal were compared each to the group normal

Results of our models adjusted for gender, age, and the interaction of gender by age at baseline revealed the following results. Pronounced self-reported mental health problems at baseline were significantly associated with impaired self-reported general and mental health 6 years later, and with impaired self-reported general, mental and physical health 11 years later (see Table 2 and Fig. 7). The self-reported impact status due to mental health problems at baseline was significantly negatively associated with general and mental health 6 years later as well as 11 years later; that is, severe self-reported impact due to mental health problems at baseline was associated with reduced general and mental health 6 and 11 years later. We further found that pronounced parent-reported mental health problems at baseline were associated with impaired self-reported general, mental and physical health 6 as well as 11 years later. However, we found no effects for the parent-reported impact of mental health problems at baseline on self-reported health outcomes at follow-ups (see Table 2 and Fig. 8). Our findings additionally showed that a higher SES was associated consistently with better general health, and with better physical health in two out of four models at follow-ups. Reported effects were consistently small (0.01 ≤ η^2^ < 0.06; see Table 2 and Figs. 7 and 8; please note, results on SES are not included in the figures).

**Table 2:** Mental health problems and their impact at baseline, and health-related outcomes at 6-year and 11-year follow-ups

	6-year follow-up			11-year follow-up		
	General health (SF-36 scale, 0–100)	Mental health (SF-36 scale, 0–100)	Physical health (SF-36 scale, 0–100)	General health (SF-36 scale, 0–100)	Mental health (SF-36 scale, 0–100)	Physical health (SF-36 scale, 0–100)
	B [95% CI]	B [95% CI]	B [95% CI]	B [95% CI]	B [95% CI]	B [95% CI]
Self-reported mental health problems at baseline (SDQ total difficulties score)	− 0.3* [− **0.7, **− **0.0]**	− 0.3*** [− **0.5, **− **0.2]**	− 0.1 [− 0.2, 0.0]	− 0.6** [− **1.0, **− **0.2]**	− 0.3** [− **0.5, **− **0.1]**	− 0.3*** [− **0.4, **− **0.1]**
Self-reported impact of mental health problems at baseline (SDQImpact score)						
[Ref. Normal]						
Borderline	− 6.4* [− **11.4, **− **1.3]**	− 3.4** [− **5.7, **− **1.0]**	− 0.8 [− 2.3, 0.8]	− 3.5 [− 9.1, 2.2]	− 0.5 [− 3.6, 2.6]	− 1.0 [− 3.1, 1.0]
Abnormal	− 10.2*** [− **15.6, **− **4.9]**	− 5.1*** [− **7.6, **− **2.6]**	− 1.0 [− 2.6, 0.6]	− 6.1* [− **12.0, **− **0.3]**	− 6.2*** [− **9.5, **− **2.9]**	− 0.3 [− 2.4, 1.8]
Socio-economic status	0.9*** [0.6, 1.3]	− 0.1 [− 0.3, 0.1]	0.1* [0.0, 0.3]	0.8*** [0.4, 1.2]	0.1 [− 0.1, 0.4]	0.1 [− 0.1, 0.2]
*Model fit* (*adjusted R*^*2*^)	*0.13*	*0.13*	*0.4*	*0.10*	*0.08*	*0.04*
Parent-reported mental health problems at baseline (SDQ total difficulties score)	− 0.6** [− **0.9, **− **0.2]**	− 0.3*** [− **0.5, **− **0.2]**	− 0.1* [− **0.2, 0.0]**	− 0.6*** [− **0.9, **− **0.3]**	− 0.2* [− **0.4, 0.0]**	− 0.2*** [− **0.3, **− **0.1]**
Parent-reported impact of mental health problems at baseline (SDQ-Impact score)						
[Ref. Normal]						
Borderline	− 3.1 [− 9.2, 3.0]	− 0.9 [− 3.8, 2.0]	− 0.4 [− 2.2, 1.4]	− 3.0 [− 8.3, 2.3]	0.3 [− 2.6, 3.2]	− 1.7 [− 3.5, 0.2]
Abnormal	− 3.5 [− 9.1, 2.1]	− 2.6 [− 5.3, 0.0]	− 1.2 [− 2.9, 0.5]	− 4.0 [− 8.5, 0.5]	− 2.2 [− 4.7, 0.3]	− 1.0 [− 2.6, 0.6]
Socio-economic status	0.9*** [0.5, 1.3]	− 0.1 [− 0.2, 0.1]	0.1 [0.0, 0.2]	0.8*** [0.4, 1.1]	0.1 [0.0, 0.3]	0.2** [0.0, 0.3]
*Model fit* (*adjusted R*^2^)	*0.11*	*0.08*	*0.04*	*0.10*	*0.06*	*0.05*

All analyses were adjusted for gender, age at baseline, and gender by age at baseline.

B unstandardized regression coefficient, CI confidence interval.

*p ≤ 0.05, **p ≤ 0.01, ***p ≤ .001. Small effects according to partial eta square (0.01 ≤ *η*^2^ < 0.06) are printed in bold. For measures see “Methods”

### Mental health care utilisation

Out of the total BELLA sample of the 11-year follow-up, 7.0% (*n* = 144) of participants aged 14 years or older reported a mental disorder, which was recently diagnosed by a physician, psychologist or other professional. A percentage of 61.8% (*n* = 89) of these mentally ill adolescents and young adults were, therefore, in mental health care, and the majority (71.9%, *n* = 64) of these patients were “rather happy” or “very happy” with the treatment. Among participants in mental health care, 37.1% (*n* = 33) rated the treatment as “very effective” and 46.1% (*n* = 41) as “a little bit effective”. A percentage of 38.2% (*n* = 55) of the participants currently used no mental health treatment, even though a mental disorder was recently diagnosed. The six most frequently mentioned reasons (multiple answers possible) for no mental health care use were no interest in treatment (*n* = 15), treatment already finished (*n* = 14), participant under medical treatment by physician (*n* = 14), poor communication with professional (*n* = 6), uncertainty about severity of the problem (*n* = 6), and fear of stigma (*n* = 5). Concerning a lifetime mental disorder, a total of 291 (14.2%) participants reported ever being diagnosed with a mental disorder, and 80.4% (*n* = 234) of these patients were in psychological, psychiatric or psychotherapeutic treatment.

Parents reported diagnoses of mental health disorders for their children aged 7 to 13 years. In total, 8.3% (*n* = 117) of parents stated that their child was recently diagnosed with a mental disorder by a physician, psychologist or other professional; 65.0% (*n* = 76) of these children were in mental health care, and the majority (77.6%, *n* = 59) of parents were “rather happy” or “very happy” with the child’s treatment. A total of 39.5% (*n* = 30) of parents with children in mental health care assessed the treatment as “very effective”, and 42.1% (*n* = 32) assessed the treatment as “a little bit effective”. A total of 35.0% (*n* = 41) of the parents reported that their child currently used no mental health treatment, even though a mental disorder was recently diagnosed. The most frequently mentioned reasons (multiple answers possible) for no mental health care use were as follows: child is under medical treatment by the physician (*n* = 18), treatment is already finished (*n* = 15), and child is under treatment by Ergo therapist (*n* = 11). Concerning a lifetime mental disorder, a total of 178 (12.7%) parents reported that their children had ever been diagnosed with a mental disorder, and 75.8% (*n* = 135) of these children were in psychological, psychiatric or psychotherapeutic treatment.

## Discussion

The BELLA study is a profound and comprehensive longitudinal study on mental health and HRQoL in children and adolescents in Germany. The BELLA study provides solid data that enable both cross-sectional and longitudinal analyses of child and adolescent mental health, mental health care use and developmental trajectories of mental health from childhood to young adulthood. The aims of the present paper were to describe the design and methods of the 11-year follow-up of the BELLA study, to examine age- and gender-specific courses of general health and HRQoL, to investigate long-term health outcomes of mental health problems and to report on mental health care use in young people in Germany.

For the 11-year follow-up of the BELLA study, we predicted study participation and investigated drop-out. We only found a considerable difference between responders and non-responders in age. Furthermore, a higher likelihood for dropout was detected in young people with a lower SES and in non-German citizens (in line with published findings from the 6-year follow-up [19]). For health- and mental health-related variables, we found only negligible effects if significance was detected at all. To compensate for sociodemographic differences, a weighting variable was generated that allows representative analyses of the cross-sectional sample.

In our longitudinal analyses on the course of general health and HRQoL from childhood via adolescence to young adulthood, we found significant differences by age and gender investigating self- and parent reports. Self-reported general health (in 10- to 31-year-olds) was better in younger than older participants and in boys compared to girls, and the age-specific difference was more pronounced in girls. Similar patterns in relation to age and gender were found for parent-reported general health (in 3- to 20-year-olds), indicating that overall health was better in younger than older participants, with higher scores for girls in younger participants and for boys in older participants. Our findings are in line with the results of a number of cross-national studies reporting that self-reported subjective health complaints increase from childhood to adolescence and are more prevalent among girls than boys [65, 66]. Furthermore, a representative epidemiological study with more than 2000 parent–adolescent dyads from Australia reported clinically significant differences in the perceptions of general health comparing child and parent reports [67]. We did not compare self- and parent reports directly, but our graphs point in a similar direction. These results may imply that adolescents are less positive about their health than their parents. In particular, girls reported worse general health, which might partly be due to “gendered” health complaints such as headache and abdominal pains, in which mid-adolescent girls had more than 2.5 higher odds than boys of reporting recurrent patterns [66]. Future epidemiological studies should be aware of the age- and gender-specific differences in general health in self- and parent-reports.

Furthermore, self- and parent-reported HRQoL was better in younger than older participants, covering an age range from 6- to 20-year-olds. This finding is in line with previous international studies that reported that younger age was significantly associated with perceptions of better overall HRQoL [68–70]. Moreover, our study findings indicate that self-reported HRQoL was better in male participants than in female participants, whereas the decrease with ongoing age was more pronounced in girls. The results are in line with the findings of previous studies, indicating that from about age 9 or 10 years on girls had lower HRQoL scores in most of the dimensions of HRQoL [68, 71, 72] and that there was a stronger decline in HRQOL among girls compared to boys with increasing age [70]. Overall, our findings on the trajectories of general health and HRQoL highlight the importance of effective prevention strategies that need to respond sensitively to age- and gender-specific differences.

Regarding the examined impact of mental health problems, we found negative long-term health outcomes of mental health problems during childhood and adolescence, confirming results from previous studies investigating this relationship [73–75]. In particular, symptom severity during childhood and adolescence predicted general health and mental health and, to a lesser extent, physical health 6–11 years later. Further, a high subjective impact of mental health problems in children and adolescents (but not the impact as perceived by their parents) predicted impaired general and mental health 6 and 11 years later. Copeland et al. [76] conducted a longitudinal study using more than 1,200 participants from an US–American population-based sample from childhood (9–16 years) to young adulthood (19–26 years of age), and they found that individuals with a childhood mental disorder had sixfold higher odds of at least one adverse adult outcome (e.g., multiple psychiatric problems) compared to those with no history of mental health problems in childhood. The results were also robust for participants without a diagnosed mental disorder but with subthreshold mental health problems and even stronger for cumulative childhood exposure to mental disorder [76]. To prevent those negative long-term consequences, the early detection of children and adolescents at risk of developing mental health problems is of great importance as it is the first step towards prevention. In this context, early intervention services such as the Australian National Youth Mental Health Foundation’s headspace [77] can support young people who experience mental health problems and help them access health services.

Mental health problems during childhood and adolescence predicted young adults’ health outcomes in the presented models. We found small effects and explained 4–13% of the variance in the outcomes in our general population sample over 6 and 11 years. It is known that mental health disorders often begin in childhood or adolescence and persist into adulthood [16]. In a former analysis based on data from the BELLA study [19] using further self- and parent-reported measures besides the SDQ, about 31% of the participants with mental health problems at baseline had corresponding problems at the 6-year follow-up (in line with Ihle and Esser [78]). Results from British cohort studies are important to consider as well, though not necessarily directly comparable. Findings from the Millenium Cohort study showed moderate stability of mental health outcomes over a period of three years analysing parent-reports for 11-year-olds and self-reports for 14-year-olds [79]. In another British study, strong stability in mental health scores was found over 3 years based on data from two measurement points (using self-, parent- and teacher-reports of the SDQ [80]). Moreover, studies analyzing trajectories of mental health problems over time usually include further factors (see e.g., this Australian study [81]). Research showed that multiple adverse childhood experiences (e.g., abuse experiences, parental separation, and growing up in a household with mental illness) increase the risk of negative health outcomes in adulthood [82–84]. Factors such as school connectedness and cognitive ability [85], bullying experiences [86], and drug abuse may additionally affect the development of mental health in young peoples’ lives. Overall, we assume that our present results reflect that we used a limited selection of predictors (measured all at baseline), health outcomes from only one follow-up measurement in each model and did not combine information from different sources. Further studies based on data of the BELLA study will aim to investigate the course of mental health problems from childhood via adolescence into adulthood in more depth considering potential risk and protective factors, data from more measurement points and different respondents in a model.

With regard to the present results, the SES of the family in childhood was a significant predictor of general health, mental health, and physical health in young adults aged 18–28 years. This finding is in line with research on social inequality and health. Children with a low SES suffer not only from greater health problems in childhood but also from poorer health outcomes in adulthood [87, 88]. To reduce the identified health inequalities, targeted and low-threshold approaches of prevention and intervention are needed, especially for children with a socially deprived background.

In our analysis regarding reported diagnoses for mental disorders and mental health care use, we were able to confirm the results of recent studies. In the total BELLA sample at the 11-year follow-up, 7.0% of self-reports and 8.2% of parent reports indicated a current diagnosis of a mental disorder. Of those with a diagnosed mental disorder, 71.5% of self-reports and 77.8% of parents reports stated that they are currently under mental health treatment or that they finished treatment. Similar results were found in the representative 2016 US-National Survey of Children’s Health, according to which up to 80.0% of participants with a mental disorder (prevalence ranged from 3.2 to 7.4%) received treatment in the previous year with differences by specific mental disorders [89]. However, our results do not provide information about access to care, quality of mental health care and treatment success, which are important determinants for health care and should be examined in future research. Since our findings revealed that approximately one out of four children with a diagnosed mental disorder is not under mental health treatment, it is important to better understand which factors inhibit or facilitate access to health care. Another important aspect is the need for an efficient transition from paediatric to adult care to prevent adolescents and young adults from dropping out of care. As the process of transition is often poorly managed, strategies are needed to ensure the successful transition to adult-oriented care [90].

## Strengths and limitations

The BELLA study is one of the most important longitudinal studies on mental health and HRQoL in children and adolescents in Germany. Our findings provide new cross-sectional as well as longitudinal data on child and adolescent mental health, HRQoL and mental health service use, which were collected nationwide across Germany. The strengths of the BELLA study include the profound and solid data, the large population-based cohort and the wide age range of the participants from 7 to 31 years. This allowed us to analyse developmental trajectories of mental health and well-being from childhood via adolescence to young adulthood. The BELLA study focuses on mental health, well-being, and young people’s resources rather than mental disorders. Thus, the findings of the BELLA study are important for the development of targeted mental health promotion and early prevention strategies. Furthermore, the BELLA study used standardised and established self- and parent-reported instruments to assess mental health, HRQoL and mental health care utilisation. Moreover, we used appropriate statistical approaches (e.g., individual growth modelling) to analyse our longitudinal data.

Despite these strengths, there are some limitations. Data were not collected in different languages; thus, families with migration backgrounds could not be treated as representative of migrant families in Germany. A further limitation and common problem of longitudinal studies is the loss to follow-up over time. However, several approaches were undertaken to compensate for loss to follow-up in the BELLA and KiGGS studies. These include the application of a weighting procedure to correct for deviations from the sociodemographic and socioeconomic structure of the target population as well as an oversampling of families with migration backgrounds. Moreover, the BELLA study is an observational study that only identifies associations and no cause–effect relationships. In our analyses, we used only single items to investigate mental health care use; future research may wish to investigate mental health care use in more depth.

## Conclusion

With the most recent 11-year follow-up, the prospective longitudinal BELLA study provides new data on mental health and HRQoL in children, adolescents, and young adults in Germany that are of great relevance for health promotion and prevention practices. The first results on mental health, HRQoL and mental health care use in children, adolescents and young adults were presented in the present paper. Future analyses using cross-sectional and longitudinal data of the BELLA study are planned, including the provision of reference scores for PROMIS instruments, reference scores and investigations on HRQoL using data collected by means of the Kids-CAT, and the development of further CATs. The investigation of mental health and well-being from childhood over adolescence into adulthood is still a challenge. In large surveys and clinical studies, we usually follow the state of the art by measuring mental health and well-being with age-appropriate questionnaires. Future research should review given statistical approaches to overcome this challenge and provide, use, and describe practical procedures that allow age comprehensive comparisons and tracking of mental health and well-being across age groups based on longitudinal data from a large survey.

## Electronic supplementary material

Below is the link to the electronic supplementary material.

Supplementary file1 (PDF 225 kb)

Supplementary file2 (PDF 106 kb)

## References

[1] Erskine HE (2015). A heavy burden on young minds: the global burden of mental and substance use disorders in children and youth. Psychol Med.

[2] World Health Organization (2017). Mental health atlas 2018.

[3] Polanczyk GV (2015). Annual research review: a meta-analysis of the worldwide prevalence of mental disorders in children and adolescents. J Child Psychol Psychiatry.

[4] Jonsson U (2017). Annual research review: quality of life and childhood mental and behavioural disorders - a critical review of the research. J Child Psychol Psychiatry.

[5] Wittchen HU (2011). The size and burden of mental disorders and other disorders of the brain in Europe 2010. Eur Neuropsychopharmacol.

[6] Belfer ML (2008). Child and adolescent mental disorders: the magnitude of the problem across the globe. J Child Psychol Psychiatry.

[7] Kieling C (2011). Child and adolescent mental health worldwide: evidence for action. Lancet.

[8] Kovess-Masfety V (2016). Comparing the prevalence of mental health problems in children 6–11 across Europe. Soc Psychiatry Psychiatr Epidemiol.

[9] Barkmann C, Schulte-Markwort M (2010). Prevalence of emotional and behavioural disorders in German children and adolescents: a meta-analysis. J Epidemiol Community Health.

[10] Schaefer JD (2017). Enduring mental health: prevalence and prediction. J Abnorm Psychol.

[11] Farmer RF (2013). Aggregation of lifetime axis i psychiatric disorders through age 30: incidence, predictors, and associated psychosocial outcomes. J Abnorm Psychol.

[12] Bastiaansen D, Koot HM, Ferdinand RF (2005). Determinants of quality of life in children with psychiatric disorders. Qual Life Res.

[13] Houtrow A, Okumura M (2011). Pediatric mental health problems and associated burden on families. Vulnerable Child Youth Stud.

[14] Breslau J (2008). Mental disorders and subsequent educational attainment in a US national sample. J Psychiatr Res.

[15] Olesen J (2012). The economic cost of brain disorders in Europe. Eur J Neurol.

[16] Kessler RC (2012). Prevalence, persistence, and sociodemographic correlates of DSM-IV disorders in the National Comorbidity Survey Replication Adolescent Supplement. Arch Gen Psychiatry.

[17] Lieb R (2016). Impact of specific phobia on the risk of onset of mental disorders: a 10-year prospective-longitudinal community study of adolescents and young adults. Depress Anxiety.

[18] Kessler RC (2005). Lifetime prevalence and age-of-onset distributions of DSM-IV disorders in the National Comorbidity Survey Replication. Arch Gen Psychiatry.

[19] Ravens-Sieberer U (2015). The longitudinal BELLA study: design, methods and first results on the course of mental health problems. Eur Child Adolesc Psychiatry.

[20] Reardon T (2017). What do parents perceive are the barriers and facilitators to accessing psychological treatment for mental health problems in children and adolescents? A systematic review of qualitative and quantitative studies. Eur Child Adolesc Psychiatry.

[21] Gulliver A, Griffiths KM, Christensen H (2010). Perceived barriers and facilitators to mental health help-seeking in young people: a systematic review. BMC Psychiatry.

[22] Ravens-Sieberer U (2008). Health-related quality of life in children and adolescents in Germany: results of the BELLA study. Eur Child Adolesc Psychiatry.

[23] Dey M, Landolt MA, Mohler-Kuo M (2012). Health-related quality of life among children with mental disorders: a systematic review. Qual Life Res.

[24] Sharpe H (2016). Exploring the relationship between quality of life and mental health problems in children: implications for measurement and practice. Eur Child Adolesc Psychiatry.

[25] Kurth BM (2007). Kinder- und Jugendgesundheitssurvey (KiGGS): Ein Überblick über Planung, Durchführung und Ergebnisse unter Berücksichtigung von Aspekten eines Qualitätsmanagements. Bundesgesundheitsblatt Gesundheitsforschung Gesundheitsschutz.

[26] Lange M (2018). KiGGS Wave 2 longitudinal component—data collection design and developments in the number of participants in the KiGGS cohort. J Health Monit.

[27] Ravens-Sieberer U (2008). Prevalence of mental health problems among children and adolescents in Germany: results of the BELLA study within the National Health Interview and Examination Survey. Eur Child Adolesc Psychiatry.

[28] Hoffman R (2018). KiGGS Wave 2 cross-sectional study—participant acquisition, response rates and representativeness. J Health Monit.

[29] The American Association for Public Opinion Research (2016) Standard definitions: final dispositions of case codes and outcome rates for surveys. AAPOR. https://www.aapor.org/AAPOR_Main/media/publications/Standard-Definitions20169theditionfinal.pdf. Accessed 07 July 2020

[30] Chen H, Cohen P, Chen S (2010). How big is a big odds ratio? Interpreting the magnitudes of odds ratios in epidemiological studies. Commun Stat Simul Comput.

[31] Lampert T (2018). Socioeconomic status and subjective social status measurement in KiGGS Wave 2. J Health Monit.

[32] Frank L (2018). Improving the inclusion and participation of children and adolescents with a migration background in KiGGS Wave 2. J Health Monit.

[33] de Bruin A, PHSJ, Nossikov A (1996) Health interview surveys: towards harmonization of methods and instruments, in WHO regional publications. European series no. 58. 1996: Copenhagen 8857196

[34] Devine J (2015). A new computerized adaptive test advancing the measurement of health-related quality of life (HRQoL) in children: the Kids-CAT. Qual Life Res J.

[35] Barthel D (2017). The validation of a computer-adaptive test (CAT) for assessing health-related quality of life in children and adolescents in a clinical sample: study design, methods and first results of the Kids-CAT study. Qual Life Res.

[36] Embretson SE, Reise SP (2000). Item response theory for psychologists.

[37] Ravens-Sieberer U (2006). and the European KIDSCREEN Group, The KIDSCREEN Questionnaires—quality of life questionnaires for children and adolescents—handbook.

[38] Ware JE, Kosinski M, Keller SD (1996). A 12-item short-form health survey—construction of scales and preliminary tests of reliability and validity. Med Care.

[39] Bullinger M, Kirchberger I, Ware J (1995). Der deutsche SF-36 Health Survey. Übersetzung und psychometrische Testung eines krankheitsübergreifenden Instrumentes zur Erfassung der gesundheitsbezogenen Lebensqualität. Zeitschrift für Gesundheitswissenschaften.

[40] Cella D (2010). The patient-reported outcomes measurement information system (PROMIS) developed and tested its first wave of adult self-reported health outcome item banks: 2005e2008. J Clin Epidemiol.

[41] Forrest CB (2012). Commentary: The patient-reported outcome measurement information system (PROMIS(R)) for children and youth: application to pediatric psychology. J Pediatr Psychol.

[42] Bevans KB (2017). Children's family experiences: development of the PROMIS((R)) pediatric family relationships measures. Qual Life Res.

[43] Devine KA (2018). PROMIS peer relationships short form: how well does self-report correlate with data from peers?. J Pediatr Psychol.

[44] Forrest CB (2014). Development of the PROMIS (R) pediatric global health (PGH-7) measure. Qual Life Res.

[45] Forrest CB (2018). Development and psychometric evaluation of the PROMIS Pediatric Life Satisfaction item banks, child-report, and parent-proxy editions. Qual Life Res.

[46] Forrest CB (2016). Concurrent validity of the PROMIS(R) pediatric global health measure. Qual Life Res.

[47] Hinchcliff M (2011). Validity of two new patient-reported outcome measures in systemic sclerosis: patient-reported outcomes measurement information system 29-item health profile and functional assessment of chronic illness therapy-dyspnea short form. Arthritis Care Res (Hoboken).

[48] Tucker CA (2020). Development of the PROMIS pediatric physical activity item banks. Phys Ther.

[49] Hays RD (2018). PROMIS((R))-29 v2.0 profile physical and mental health summary scores. Qual Life Res.

[50] Devine J (2018). Translation and cross-cultural adaptation of eight pediatric PROMIS (R) item banks into Spanish and German (vol 27, pg 2415, 2018). Qual Life Res.

[51] Goodman R (1997). The Strengths and Difficulties Questionnaire: A research note. J Child Psychol Psychiatry.

[52] Goodman R (1999). The extended version of the Strengths and Difficulties Questionnaire as a guide to child psychiatric caseness and consequent burden. J Child Psychol Psychiatry.

[53] Wittchen HU (1999). Screening for mental disorders: performance of the composite international diagnostic-screener (CID-S). Int J Methods Psychiatr Res.

[54] Klaghofer R, Braehler E (2001). Konstruktion und teststatistische Prüfung einer Kurzform der SCL-90-R. Zeitschrift für Klinische Psychologie, Psychiatrie und Psychotherapie.

[55] Radloff LS (1977). The CES-D Scale: a self-report depression scale for research in the general population. Appl Psychol Meas.

[56] Kroenke K, Spitzer RL (2002). The PHQ-9: a new depression diagnostic and severity measure. Psychiatr Ann.

[57] Kroenke K (2009). The PHQ-8 as a measure of current depression in the general population. J Affect Disord.

[58] Pilkonis PA (2011). Item banks for measuring emotional distress from the patient-reported outcomes measurement information system (PROMIS®): depression, anxiety, and anger. Assessment.

[59] Wahl I, Löwe B, Rose M (2011). Das patient-reported outcomes measurement information system (PROMIS): übersetzung der item-banken für depressivität und angst ins deutsche. Klinische Diagnostik und Evaluation.

[60] Barkmann C (2008). The German version of the Centre for Epidemiological Studies Depression Scale for Children: psychometric evaluation in a population—based survey of 7–17 years old children and adolescents- results of the BELLA study. Eur Child Adolesc Psychiatry.

[61] Gräfe K, Herzog W, Löwe B (2004). Screening psychischer Störungen mit dem Gesundheitsfragebogen für Patienten (PHQ-D). Diagnostica.

[62] Lorah J (2018). Effect size measures for multilevel models: definition, interpretation, and TIMSS example. Large-scale Assess Educ.

[63] McFadden D, Zarembka P (1973). Conditional logit analysis of qualitative choice behavior. Frontiers in econometrics.

[64] Heck RH, Thomas SL, Tabata LN (2012). Multilevel modeling of categorical outcomes using IBM SPSS.

[65] Haugland S (2001). Subjective health complaints in adolescence. A cross-national comparison of prevalence and dimensionality. Eur J Public Health.

[66] Torsheim T (2006). Cross-national variation of gender differences in adolescent subjective health in Europe and North America. Soc Sci Med.

[67] Waters E, Stewart-Brown S, Fitzpatrick R (2003). Agreement between adolescent self-report and parent reports of health and well-being: results of an epidemiological study. Child Care Health Dev.

[68] Meade T, Dowswell E (2015). Health-related quality of life in a sample of Australian adolescents: gender and age comparison. Qual Life Res.

[69] Freire T, Ferreira G (2018). Health-related quality of life of adolescents: relations with positive and negative psychological dimensions. Int J Adoles Youth.

[70] Michel G (2009). Age and gender differences in health-related quality of life of children and adolescents in Europe: a multilevel analysis. Qual Life Res.

[71] Freire T, Ferreira G (2018). Health-related quality of life of adolescents: Relations with positive and negative psychological dimensions. Int J Adolesc Youth.

[72] Gonzalez EA (2016). Gender differences in health-related quality of life of Chilean adolescent students. Rev Med Chil.

[73] Fergusson DM, John-Horwood L, Ridder EM (2005). Show me the child at seven: the consequences of conduct problems in childhood for psychosocial functioning in adulthood. J Child Psychol Psychiatry..

[74] Steinhausen H-C, Winkler-Metzke CW (2004). The impact of suicidal ideation in preadolescence, adolescence, and young adulthood on psychosocial functioning and psychopathology in young adulthood. Acta Psychiatr Scand.

[75] Copeland WE (2013). Diagnostic transitions from childhood to adolescence to early adulthood. J Child Psychol Psychiatry.

[76] Copeland WE (2015). Adult functional outcomes of common childhood psychiatric problems: a prospective longitudinal study. JAMA Psychiatry.

[77] McGorry PD (2007). headspace: Australia's National Youth Mental Health Foundation—where young minds come first. Med J Aust.

[78] Ihle W, Esser G (2002). Epidemiologie psychischer Störungen im Kindes- und Jugendalter: Prävalenz, Verlauf, Komorbidität und Geschlechtsunterschiede. Psychol Rundsch.

[79] Patalay P, Fitzsimons E (2018). Development and predictors of mental ill-health and wellbeing from childhood to adolescence. Soc Psychiatry Psychiatr Epidemiol.

[80] Ford T (2007). A prospective study of childhood psychopathology: independent predictors of change over three years. Soc Psychiatry Psychiatr Epidemiol.

[81] Christensen D (2017). Longitudinal trajectories of mental health in Australian children aged 4–5 to 14–15 years. PLoS ONE.

[82] Heidinger LS, Willson AE (2019). The childhood roots of adult psychological distress: Interdisciplinary perspectives toward a better understanding of exposure to cumulative childhood adversity. Child Abuse Negl.

[83] Hughes K (2016). Relationships between adverse childhood experiences and adult mental well-being: results from an English national household survey. BMC Public Health.

[84] Kalmakis KA, Chandler GE (2015). Health consequences of adverse childhood experiences: a systematic review. J Am Assoc Nurse Pract.

[85] Patalay P, Fitzsimons E (2016). Correlates of mental illness and wellbeing in children: are they the same? Results from the UK Millennium Cohort Study. J Am Acad Child Adolesc Psychiatry.

[86] Lereya ST (2015). Bully/victims: a longitudinal, population-based cohort study of their mental health. Eur Child Adolesc Psychiatry.

[87] Conroy K, Sandel M, Zuckerman B (2010). Poverty grown up: how childhood socioeconomic status impacts adult health. J Dev Behav Pediatr.

[88] Cohen S, Adler NE, Stewart J (2010). Childhood socioeconomic status and adult health. Biology of disadvantage: socioeconomic status and health.

[89] Ghandour RM (2019). Prevalence and treatment of depression, anxiety, and conduct problems in US children. J Pediatr.

[90] Crowley R (2011). Improving the transition between paediatric and adult healthcare: a systematic review. Arch Dis Child.

