# Supplementary material for: Mental health and well-being from childhood to adulthood: design, methods and results of the 11-year follow-up of the BELLA study
Source: Eur Child Adolesc Psychiatry. 2020 Sep 12;30(10):1559–77. doi: 10.1007/s00787-020-01630-4 (PMC8505294; doi:10.1007/s00787-020-01630-4)
Supplement: Supplementary file 1 — Supplementary file1 (PDF 225 kb) [file 787_2020_1630_MOESM1_ESM.pdf]

Supplementary Material

File 1

Mental health and well-being from childhood to adulthood: Design, methods and results of the 11-year follow-up of the BELLA study

Journal: Journal of European Child and Adolescent Psychiatry

Authors: Christiane Otto*, Franziska Reiss*, Catharina Voss, Anne Wüstner, Ann-Katrin Meyrose, Heike Hölling & Ulrike Ravens-Sieberer

*both authors contributed equally to this manuscript (shared first authorship)

Corresponding author: Ulrike Ravens-Sieberer, Department of Child and Adolescent Psychiatry, Psychotherapy, and Psychosomatics, University Medical Center Hamburg-Eppendorf, Martinistr. 52, 20246 Hamburg, Germany, E-mail: ravens-sieberer@uke.de

Supplementary Table S1 Age of participants at the measurement points of the BELLA study

	BELLA baseline cohort					New participants (included at the 6-year follow-up)		New participants (11-year follow-up)
**Age of participants**	**Baseline (2002-2006)**	**1-year follow-up (2004-2007)**	**2-year follow-up (2005-2008)**	**6-year follow-up (2009-2012)**	**11-year follow-up (2014-2017)**	**6-year follow-up (2009-2012)**	**11-year follow-up (2014-2017)**	**11-year follow-up (2014-2017)**
3 years						47		
4 years						128		
5 years						152		
6 years						202		
7 years	268					256	1	51
8 years	283	234				197	3	112
9 years	287	249	206			187	9	144
10 years	291	250	231			151	9	135
11 years	265	250	240			110	82	165
12 years	252	212	225			110	113	168
13 years	261	219	181	28		91	121	181
14 years	221	227	207	117		91	126	172
15 years	266	183	208	167		88	75	146
16 years	243	220	167	166		67	60	133
17 years	226	191	192	130	3	61	58	144
18 years		188	158	159	65	72	54	27
19 years			175	111	85	66	38	2
20 years				127	100	73	35	
21 years				95	109	68	24	
22 years				105	82	56	29	
23 years				224	91	138	40	
24 years					90		39	
25 years					81		40	
26 years					78		20	
27 years					62		30	
28 years					71		27	
29 years					37		12	
30 years					15		4	
31 years					4		1	
Total	***2,863***	***2,423***	***2,190***	***1,429***	***973***	***2,411***	***1,050***	***1,580***

Supplementary Table S2 Measurements used at the measurement points of the BELLA study

Construct	Standardised measures/specific items	Baseline	1-year follow-up	2-year follow-up	6-year follow-up	11-year follow-up
**Socio-demographic Variables**						
Gender	single item	✓	✓	✓	✓	✓
Age	single item	✓	✓	✓	✓	✓
Socio-economic status	Winkler-Index[1]	✓	-	-	-	-
	Revised SES Index[2]	-	-	-	✓	✓
**Mental Health Problems**						
General mental health problems	SDQ	✓	✓	✓	✓	✓
Anxiety	SCARED	✓	✓	✓	✓	-
	PHQ-Screener	-	-	-	✓	✓
	Selected items of the FBB-ANG assessing Anxiety disorders (from the DISYPS-KJ)	-	-	✓	-	-
Depression	CES-DC	✓	✓	✓	✓	✓
	ADS	-	-	-	-	✓
	DIKJ	✓	✓	✓	-	-
	PHQ-8	-	-	-	✓	✓
	Selected items of the FBB-DES assessing Depressive disorders (from the DISYPS-KJ)	-	-	✓	-	-
	PROMIS-Depression (short form)	-	-	-	-	✓
Conduct Disorder	CBCL	✓	✓	✓	✓	-
ADHD	CRS-R	✓	✓	✓	✓	-
	FBB-HKS assessing Hyperkinetic disorders (from the DISYPS-KJ)	✓	✓	✓	-	-
Eating Disorders	Body weight in kilograms and height in meter for calculating the Body Mass Index (BMI)	✓	✓	✓	✓	✓
**Mental Disorders**						
	SCOFF	✓	✓	✓	✓	✓
	Confirmed diagnosis of mental health problems by physician.	✓	✓	✓	✓	✓
	DIPS for Anxiety Disorders, Affective Disorders, Conduct Disorders, ADHD, Eating Disorders, Substance abuse/addiction.	-	-	-	✓	-
	CID-S	-	-	-	✓	✓
**Health-related Quality of Life**						
	Kids-CAT	-	-	-	-	✓
	KIDSCREEN	✓	✓	✓	✓	✓
	KINDL-R	✓	✓	✓	✓	-
	SF-12	-	-	-	-	✓
	SF-36	-	-	-	✓	✓
	PROMIS-Subjective well-being	-	-	-	-	✓
	PROMIS-Family well-being	-	-	-	-	✓

	PROMIS-Physical activity	-	-	-	-	✓
	PROMIS-Relations with peers	-	-	-	-	✓
	PROMIS-General health	-	-	-	-	✓
	PROMIS-Profile 29	-	-	-	-	✓
**Risk factors**						
Parental psychopathology	SCL-S-9	✓	✓	✓	✓	-
Parental quality of life	SF-12	✓	✓	✓	✓	-
Parental strain	Items assessing the burden caused by housekeeping, being a single parent, tending a family member in need of care, job-related problems, or financial problems	✓	-	-	✓	✓
Risk-Index	Items assessing family conflicts, harmony in partnership, unemployment, parental chronic diseases, unwanted pregnancy, parental alcohol consumption	✓	✓	✓	✓	-
Life events	Items assessing serious illness or accident, death of a close person, marriage or new partnership, change of school	-	✓	✓	✓	✓
**Protective factors**						
Self-efficacy	GSE	✓	✓	✓	✓	✓
Self-concept	SPPC	✓	✓	✓	-	-
	Selected items of the CHIP-AE	✓	-	-	-	-
Coping	ACOPE, ECOPE	-	✓	✓	✓	-
Optimism	BFW, CSOS	✓	✓	✓	✓	✓
Family climate	FCS	✓	✓	✓	✓	✓
Parental support	8 items from the HBSC	✓	✓	✓	✓	-
Social support	8 selected age-appropriate items from the German SSS	✓	✓	✓	✓	✓
Peer competence	5 items from the HBSC	✓	✓	✓	✓	-
School climate	5 items from the HBSC	✓	✓	✓	✓	-
**Health care utilisation**						
	Items assessing use (and frequency of use) of health care utilisation by professionals, e.g. psychiatrist or psychologist	✓	✓	✓	✓	✓
	Items assessing paths of mental health care utilisation	-	-	-	✓	-
	Items assessing knowledge of mental health care provision	-	-	-	✓	✓
	Items assessing satisfaction with health care services	-	-	-	✓	✓
	Items assessing barriers towards mental health care utilisation	-	-	-	✓	✓

[1] by Winkler & Stolzenberg [1]; [2] by Lampert et al. [2, 3]

Note. Explanations of abbreviations and references are presented at the end of this file.

List of abbreviations

ACOPE	Problem-focused Coping [Problemorientiertes, aktives Coping] [4]
ADHS	Attention deficit hyperactivity disorder
BFW	Berner Questionnaire on Adolescents' Subjective Well-Being [Berner Fragebogen für Wohlbefinden] [5]
CBCL	Child Behavior Checklist [6]; German version by Arbeitsgruppe Deutsche Child Behavior Checklist [7]
CES-DC	Center for Epidemiological Studies Depression Scale for Children [8]; German version by Barkmann et al. [9]
CHIP-AE	Child Health and Illness Profile-Adolescent Edition [10]
CID-S	Composite International Diagnostic Screener [11]
CRS-R	Conners‘ Rating Scales-Revised [12]; German version by Erhart, Döpfner [13]
CSOS	Children's Sense of Coherence Scale [14]; German version by Kern [15]
DIKJ	Depression Inventory for Children and Adolescents [Depressions-Inventar für Kinder und Jugendliche] [16, 17]
DIPS	Diagnostic Interview for Mental Disorders in Children and Adolescents [Diagnostisches Interview bei psychischen Störungen im Kindes- und Jugendalter] [18, 19]
DISYPS-KJ	Diagnostic System for Mental Disorders in Childhood and Adolescence [Diagnostik-System für psychische Störungen im Kindes- und Jugendalter] [20]
ECOPE	Emotion-focused Coping [Emotionsorientiertes, vermeidendes Coping] [21]
FBB-ANG	Proxy-questionnaire for Anxiety disorders [Fremdbeurteilungsbogen für Angststörungen] from the Diagnostic System for Mental Disorders in Childhood and Adolescence [Diagnostik-System für psychische Störungen im Kindes- und Jugendalter] [20]
FBB-DES	Proxy-questionnaire for Depressive disorders [Fremdbeurteilungsbogen für Depressive Störungen]) from the Diagnostic System for Mental Disorders in Childhood and Adolescence [Diagnostik-System für psychische Störungen im Kindes- und Jugendalter] [20]
FBB-HKS	Proxy-questionnaire for Hyperkinetic disorders [Fremdbeurteilungsbogen für hyperkinetische Störungen] from the Diagnostic System for Mental Disorders in Childhood and Adolescence [Diagnostik-System für psychische Störungen im Kindes- und Jugendalter] [20]
FCS	Family Climate Scale [22], which is the German adaptation of the Family Environmental Scale [23, 24]
GSE	General Self-Efficacy Scale [25]; German version by Bäßler and Schwarzer [21]
HBSC	Health Behaviour in School-aged Children survey [26]
KIDS-CAT	Kids-Computer-Adaptive Test [27]
KIDSCREEN	Quality of Life Questionnaire for Children and Adolescents [28]
KINDL-R	Questionnaire to assess Health-related Quality of Life in Children and Adolescents – Revised version [29]
PHQ-8	Patient Health Questionnaire-8 [30]; German version by Löwe, Spitzer [31] and Gräfe, Zipfel [32]
PHQ-Screener	Patient Health Questionnaire-Screener for Panic Syndrom [33]; German version by Löwe, Spitzer [31] and Gräfe, Zipfel [32]
PROMIS	Patient-Reported Outcomes Measurement Information System [34, 35]
SCARED	Screen for Child Anxiety Related Emotional Disorders [36, 37]; German version by Plass, Barkmann [38]
SCL-S-9	Symptom-Checklist Shortversion-9 [39]
SCOFF	SCOFF Questionnaire [40], German version by Hölling and Schlack [41]
SDQ	Strengths and Difficulties Questionnaire [42] & Impact Supplement [43]
SES	socio-economic status (parental education, occupational qualification, occupational position, income)
SF-12	Short Form-12 Health Survey [44]; German version by Bullinger and Kirchberger [45]
SF-36	Short Form-36 Health Survey [46]; German version by Bullinger and Kirchberger [45]
SPPC	Self-Perception Profile for Children [47]; German version by Asendorpf and Aken [48]
SSS	Social Support Scale [49]

Supplementary References

1. Winkler, J. and H. Stolzenberg, [Social class index in the Federal Health Survey]. Gesundheitswesen, 1999. 61 Spec No: p. S178-83.
2. Lampert, T., et al., Messung des soziookonomischen Status in der Studie zur Gesundheit Erwachsener in Deutschland (DEGS1). Bundesgesundheitsblatt Gesundheitsforschung Gesundheitsschutz, 2013. 56(5-6): p. 631-6.
3. Lampert, T., et al., Messung des sozioökonomischen Status und des subjektiven sozialen Status in KiGGS Welle 2. Journal of Health Monitoring, 2018. 3(1): p. 114-133.
4. Jerusalem, M. and W. Mittag, Problemorientiertes, aktives Coping (ACOPE), in Skalen zur Erfassung von Lehrer- und Schülermerkmalen. Dokumentation der psychometrischen Verfahren im Rahmen der Wissenschaftlichen Begleitung des Modellversuchs Selbstwirksame Schulen, R. Schwarzer and M. Jerusalem, Editors. 1999, Freie Universität Berlin, Institut für Psychologie: Berlin. p. 26-27.
5. Grob, A., et al., Berner Fragebogen zum Wohlbefinden Jugendlicher (BFW). Diagnostica, 1991. 37(1): p. 66-75.
6. Achenbach, T.M., Manual for the Child Behavior Checklist/4–18 and 1991 profile. 1991, Burlington: University of Vermont, Department of Psychiatry.
7. Arbeitsgruppe Deutsche Child Behavior Checklist, Elternfragebogen über das Verhalten von Kindern und Jugendlichen; deutsche Bearbeitung der Child Behavior Checklist (CBCL/4-18). Einführung und Anleitung zur Handauswertung mit deutschen Normen. Vol. 2. 1998, Köln: Arbeitsgruppe Kinder-, Jugend- und Familiendiagnostik (KJFD).
8. Weissman, M.M., H. Orvaschel, and N. Padian, Children's symptom and social functioning self-report scales comparison of mothers' and children's reports. Journal of Nervous & Mental Disease, 1980. 168(12): p. 736-740.
9. Barkmann, C., et al., The German version of the Centre for Epidemiological Studies Depression Scale for Children: Psychometric evaluation in a population - based survey of 7 to 17 years old children and adolescents- results of the BELLA study. European Child & Adolescent Psychiatry, 2008. 17(1): p. 116-124.
10. Starfield, B., et al., Manual for the Child Health and Illness Profile-Adolescent Edition (CHIP-AE). 2000, Baltimore, MD: The Johns Hopkins University.
11. Wittchen, H.U., et al., Screening for mental disorders: Performance of the Composite International Diagnostic – Screener (CID–S). Int J Methods Psychiatr Res, 1999. 8(2): p. 59-70.
12. Lidzba, K., H. Christiansen, and R. Drechsler, Conners 3 - Conners Skalen zu Aufmerksamkeit und Verhalten 3 (Manual). 3 ed. 2013, Bern: Huber.
13. Erhart, M., M. Döpfner, and U. Ravens-Sieberer, Psychometric properties of two ADHD questionnaires: comparing the Conners' scale and the FBB-HKS in the general population of German children and adolescents- results of the BELLA study. European Child & Adolescent Psychiatry, 2008. 17 (Suppl. 1): p. 106-115.
14. Margalit, M. and M. Efrati, Loneliness, coherence and companionship among children with learning disorders. Educational Psychology, 1996. 16(1): p. 69-79.
15. Kern, R., E. Rasky, and R. Noack, Indikatoren für Gesundheitsförderung in der Volksschule. 1995, Graz: Karl-Franzens-Universität.
16. Stiensmeier-Pelster, J., M. Schürmann, and K. Duda, DIKJ- Depressions- Inventar für Kinder und Jugendliche. 2., überarbeitete und neunormierte Auflage. 2000, Göttingen: Hogrefe.
17. Stiensmeier-Pelster, J., M. Schürmann, and K. Duda, Depressions-Inventar für Kinder und Jugendliche (DIKJ). 1989, Göttingen: Hogrefe.
18. Schneider, S. and J. Margraf, DIPS: Diagnostisches Interview bei psychischen Störungen, ed. S. Schneider and J. Margraf. Vol. 4. 2011, Berlin Heidelberg: Springer.
19. Schneider, S., S. Unnewehr, and J. Margraf, Kinder-DIPS: Diagnostisches Interview bei psychischen Störungen im Kindes- und Jugendalter, ed. S. Schneider, S. Unnewehr, and J. Margraf. Vol. 2. 2009, Berlin Heidelberg: Springer.
20. Döpfner, M. and G. Lehmkuhl, Diagnostik-System für Psychische Störungen im Kindes- und Jugendalter nach ICD-10 und DSM-IV (DISYPS-KJ). 2 ed. 2000, Bern: Huber.
21. Bäßler, J. and R. Schwarzer, Emotionsorientiertes, vermeidendes Coping (ECOPE), in Skalen zur Erfassung von Lehrer- und Schülermerkmalen. Dokumentation der psychometrischen Verfahren im Rahmen der Wissenschaftlichen Begleitung des Modellversuchs Selbstwirksame Schulen, R. Schwarzer and M. Jerusalem, Editors. 1999, Freie Universität Berlin, Institut für Psychologie: Berlin. p. 27-28.
22. Schneewind, K., M. Beckmann, and A. Hecht-Jackl, Familienklima-Skalen. Bericht 8.1 und 8.2. 1985, München: Ludwig Maximilians Universität, Institut für Psychologie–Persönlichkeitspsychologie und Psychodiagnostik.

23. Moos, R.H. and B.S. Moos, Family environment scale (FES). 1974, Palo Alto, CA: Consulting Psychologists Press.
24. Moos, R.H. and B.S. Moos, Family environment scale manual and sampler set: Development, applications and research. 2009, Palo Alto, CA: Mind Garden Inc.
25. Schwarzer, R. and M. Jerusalem, Generalized Self-Efficacy Scale, in Measures in health psychology: A user's portfolio. Causal and control beliefs J. Weinman, S. Wright, and M. Johnston, Editors. 1995, NFER-NELSON: Windsor, UK. p. 35-37.
26. Currie, C., et al., Health Behaviour in School-aged Children (HBSC) study protocol: Background, methodology and mandatory items for the 2009/10 survey. 2010, Edinburgh & Vienna: CAHRU & LBIHPR.
27. Devine, J., et al., A new computerized adaptive test advancing the measurement of health-related quality of life (HRQoL) in children: the Kids-CAT. Quality of Life Research Journal, 2015. 24(4): p. 871-84.
28. Ravens-Sieberer, U. and t.E.K. Group, The KIDSCREEN Questionnaires - Quality of life questionnaires for children and adolescents – Handbook. 2006, Lengerich: Pabst Science Publisher.
29. Ravens-Sieberer, U. and M. Bullinger, Assessing health-related quality of life in chronically ill children with the German KINDL: First psychometric and content analytical results. Quality of Life Research, 1998. 7(5): p. 399-407.
30. Kroenke, K. and R.L. Spitzer, The PHQ-9: A new depression diagnostic and severity measure. Psychiatric Annals, 2002. 32(9): p. 509-515.
31. Löwe, B., et al., Gesundheitsfragebogen für Patienten (PHQ-D). Manual-Komplettversion und Kurzform. Autorisierte deutsche Version des "Prime MD Patient Health Questionnaire (PHQ). 2002, Heidelberg: Pfizer.
32. Gräfe, K., et al., Screening psychischer Störungen mit dem "Gesundheitsfragebogen für Patienten (PHQ-D)". Diagnostica, 2004. 50(4): p. 171-181.
33. Spitzer, R.L., J.B. Williams, and K. Kroenke, Instruction manual: Instructions for Patient Health Questionnaire (PHQ) and GAD-7 Measures. Retrieved from http://www.phqscreeners.com/instructions/instructions.pdf 2013.
34. Cella, D., et al., The Patient-Reported Outcomes Measurement Information System (PROMIS) developed and tested its first wave of adult self-reported health outcome item banks: 2005-2008. Journal of Clinical Epidemiology, 2010. 63: p. 1179-1194.
35. Forrest, C.B., et al., Concurrent validity of the PROMIS® pediatric global health measure. Quality of Life Research Journal, 2016. 25(3): p. 739-51.
36. Birmaher, B., et al., The Screen for Child Anxiety Related Emotional Disorders (SCARED): Scale construction and psychometric characteristics. Journal of the American Academy of Child and Adolescent Psychiatry, 1997. 36(4): p. 545-553.
37. Birmaher, B., et al., Psychometric properties of the Screen for Child Anxiety Related Emotional Disorders (SCARED): A replication study. Journal of the American Academy of Child and Adolescent Psychiatry, 1999. 38(10): p. 1230-1236.
38. Plass, A., et al., German translation and validation of the Screen for Child Anxiety Related Emotional Disorders (SCARED) - First results., in In Book of abstracts of the 16th World Congress of the International Association for Child and Adolescent Psychiatry and Allied Professions (IACAPAP). 2004: Berlin. p. 26.
39. Klaghofer, R. and E. Braehler, Konstruktion und teststatistische Prüfung einer Kurzform der SCL-90-R. Zeitschrift für Klinische Psychologie, Psychiatrie und Psychotherapie, 2001. 49(2): p. 115-124.
40. Morgan, J.F., F. Reid, and J.H. Lacey, The SCOFF questionnaire: Assessment of a new screening tool for eating disorders. Bmj, 1999. 319(1467): p. 1467-1468.
41. Hölling, H. and R. Schlack, Essstörungen im Kindes und Jugendalter - Erste Ergebnisse aus dem Kinder- und Jugendgesundheitssurvey (KiGGS). Bundesgesundheitsblatt, Gesundheitsforschung, Gesundheitsschutz, 2007. 50(5/6): p. 794-799.
42. Goodman, R., The Strengths and Difficulties Questionnaire: A research note. Journal of Child Psychology and Psychiatry, 1997. 38(5): p. 581-586.
43. Goodman, R., The extended version of the Strengths and Difficulties Questionnaire as a guide to child psychiatric caseness and consequent burden. Journal of Child Psychology and Psychiatry, 1999. 40(5): p. 791-799.
44. Ware, J.J., M. Kosinski, and S.D. Keller, A 12-Item Short-Form Health Survey: Construction of scales and preliminary tests of reliability and validity. Medical care, 1996. 34(3): p. 220-33.
45. Bullinger, M. and I. Kirchberger, SF-36 Fragebogen zum Gesundheitszustand – Handanweisung, in Diagnostische Verfahren in der Rehabilitation, J. Bengel, M. Wirtz, and C. Zwingmann, Editors. 1998, Hogrefe: Göttingen.
46. Ware, J.E. and C.D. Sherbourne, The MOS 36-Item Short-Form Health Survey (SF-36). Medical Care, 1992. 30(6): p. 473-483.

47. Harter, S., Manual for the self-perception profile for children. 1985, Denver, CO: University of Denver.
48. Asendorpf, J. and M.A.G.v. Aken, Self-Perception Profile for Children - deutsche Fassung (SPPC-D). 1993, Berlin: Humboldt-Universität, Institut für Psychologie.
49. Donald, C.A. and J.E. Ware, The measurement of social support. Research in Community & Mental Health, 1984. 4: p. 325-370.
