# Supplementary material for: Mental health and well-being from childhood to adulthood: design, methods and results of the 11-year follow-up of the BELLA study
Source: Eur Child Adolesc Psychiatry. 2020 Sep 12;30(10):1559–77. doi: 10.1007/s00787-020-01630-4 (PMC8505294; doi:10.1007/s00787-020-01630-4)
Supplement: Supplementary file 2 — Supplementary file2 (PDF 106 kb) [file 787_2020_1630_MOESM2_ESM.pdf]

Supplementary Material

File 2

Mental health and well-being from childhood to adulthood: Design, methods and results of the 11-year follow-up of the BELLA study

Journal: Journal of European Child and Adolescent Psychiatry

Authors: Christiane Otto*, Franziska Reiss*, Catharina Voss, Anne Wüstner, Ann-Katrin Meyrose, Heike Hölling & Ulrike Ravens-Sieberer

*both authors contributed equally to this manuscript (shared first authorship)

Corresponding author: Ulrike Ravens-Sieberer, Department of Child and Adolescent Psychiatry, Psychotherapy, and Psychosomatics, University Medical Center Hamburg-Eppendorf, Martinistr. 52, 20246 Hamburg, Germany, E-mail: ravens-sieberer@uke.de

Supplementary Table S3 Model fit of null models on self- and parent-reported general health and health-related quality of life

	**General health**		**Health-related quality of life**	
	**Self-report** (n = 4,987)	**parent-report** (*n* = 5,754)	**self-report** (*n* = 4,293)	**parent-report** (*n* = 4,345)
Null model fit				
-2 Log Likelihood	22,852.13	25,127.03	52,031.22	49,585.73
df	3	3	3	3
AIC	22,858.13	25,133.03	52,037.22	49,591.73
BIC	22,879.83	25,154.98	52,057.84	49,612.20

Note: analysed longitudinal data was gathered at five measurement points, only for parent-reported health-related quality of life no baseline data was available.
CI = Confidence Interval; df = degrees of freedom; AIC = Akaike's Information Criterion; BIC = Schwarz's Bayesian Information Criterion.

Supplementary Table S4 Age- and gender-specific effects on self- and parent-reported general health and health-related quality of life over time

	**General health**				**Health-related quality of life**			
	**Self-report** (n = 4,987)		**parent-report** (*n* = 5,754)		**self-report** (*n* = 4,293)		**parent-report** (*n* = 4,345)	
	b	*95 % CI*	*b*	*95 % CI*	*b*	*95 % CI*	*b*	*95 % CI*
Fixed effects								
Intercept	2.80***	2.75, 2.84	2.87***	2.83, 2.90	53.37***	52.73, 54.02	53.55***	53.01, 54.10
Level 1-predictors								
Time	0.13***	0.10, 0.17	0.09***	0.06, 0.12	2.31***	1.86, 2.77	0.81**	0.23, 1.39
Time x time	-0.03***	-0.04, -0.03	-0.03***	-0.04, -0.02	-0.83***	-0.95, -0.71	-0.54***	-0.72, -0.37
Time x time x time	0.00***	0.00, 0.00	0.00***	0.00, 0.00	0.05***	0.04, 0.06	0.05***	0.03, 0.06
Level 2-predictors								
Age	-0.01**	-0.01, -0.00	-0.03***	-0.03, -0.02	-0.78***	-0.90, -0.66	-0.27***	-0.36, -0.18
Female	-0.26***	-0.31, -0.22	-0.08***	-0.13, -0.04	-3.01***	-3.78, -2.23	-1.01**	-1.67, -0.35
Age by female	-0.01***	-0.02, -0.01	-0.01***	-0.02, -0.01	-0.11*	-0.22, -0.01	-0.16***	-0.26, -0.07
Random effects								
Residual	0.36***	0.34, 0.38	0.35***	0.34, 0.36	48.40***	45.88, 51.05	45.76***	43.13, 48.54
Intercept (subject id)	0.28***	0.25, 0.31	0.32***	0.29, 0.35	62.01***	55.64, 69.10	63.05***	57.49, 69.15
Slope (time)	0.00***	0.00, 0.00	0.00***	0.00, 0.00	0.23	0.08, 0.69	0.86***	0.60, 1.24
Intercept, slope	-0.01***	-0.02, -0.01	-0.02***	-0.02, -0.01	-3.30***	-4.71, -1.90	0.86***	0.60, 1.24
Model fit								
-2 Log Likelihood	22,515.76		24,850.33		51,401.91		49,296.58	
df	11		11		11		11	
AIC	22,537.76		24,872.33		51,423.91		49,318.58	
BIC	22,617.30		24,952.84		51,499.52		49,393.62	

Note: analysed longitudinal data was gathered at five measurement points, only for parent-reported health-related quality of life no baseline data was available.
CI = Confidence Interval; df = degrees of freedom; AIC = Akaike's Information Criterion; BIC = Schwarz's Bayesian Information Criterion.
